# Dysregulated Proline Metabolism Exacerbates Hepatocellular Carcinoma Metastasis via EPRS1-Mediated mRNA Translation

**DOI:** 10.34133/cancomm.0028

**Published:** 2026-05-14

**Authors:** Hui Zhang, Leirong Gu, Xiameng Su, Wanjin Chen, Ming Tan, Haibo Yu, Hongzhong Zhou, Tingting Gao, Zhiling Wang, Xinyan Chen, Weixian Chen, Juan Chen, Shengtao Cheng

**Affiliations:** ^1^ Department of Laboratory Medicine, Key Laboratory of Clinical Laboratory Diagnostics (Ministry of Education), The Second Affiliated Hospital, Chongqing Medical University, Chongqing, P. R. China.; ^2^ Department of Infectious Diseases, Key Laboratory of Molecular Biology for Infectious Diseases (Ministry of Education), Institute for Viral Hepatitis, The Second Affiliated Hospital, Chongqing Medical University, Chongqing, P. R. China.; ^3^ Department of Laboratory Medicine, Sichuan Provincial People’s Hospital, University of Electronic Science and Technology of China, Chengdu, Sichuan, P. R. China.; ^4^ Department of Laboratory Medicine, Shenzhen Institute of Translational Medicine, The First Affiliated Hospital of Shenzhen University, Shenzhen Second People’s Hospital, Health Science Center, Shenzhen University, Shenzhen, Guangdong, P. R. China.

## Abstract

**Background:** Metabolic reprogramming is a hallmark of hepatocellular carcinoma (HCC), and amino acid reprogramming plays an important role in its metastatic progression. However, the function of amino acid reprogramming in HCC metastatic progression remains unclear. This study aimed to elucidate the function and mechanism of amino acid reprogramming, particularly focusing on proline metabolism, in driving HCC metastasis. **Methods:** HCC cohort and multiple HCC models were enrolled to investigate the role of amino acid reprogramming in HCC metastatic progression. The pro-metastatic effect of l-proline in HCC was consistently validated across in vitro and in vivo studies. Ribosome profiling (Ribo-seq) and l-proline pull-down were used to investigate the mechanism of proline-mediated HCC metastasis. **Results:** Based on metabolomics and proteomics, we found that proline metabolism was enhanced during HCC metastasis, as evidenced by increased proline levels and proline metabolism-related enzymes in both HCC metastatic patients and mice. Moreover, high levels of proline were found to promote HCC metastasis. Mechanistically, l-proline directly interacted with glutamyl-prolyl-tRNA synthetase 1 (EPRS1) at its Glu^1123^ residue, thereby selectively enhancing the translation of phosphatidylinositol-4,5-bisphosphate 3-kinase catalytic subunit α (PIK3CA), AKT serine/threonine kinase 3 (AKT3), and integrin subunit β1 (ITGB1). Importantly, the enhanced protein translation could be abolished by bersiporocin, an EPRS1 inhibitor that prevents l-proline binding to EPRS1. Finally, the inhibitory effect of bersiporocin on HCC metastasis was confirmed in patient-derived orthotopic xenograft models. **Conclusions:** Our findings not only revealed the critical role of l-proline-bound EPRS1 in promoting HCC metastasis but also indicated that inhibiting this binding could be a promising strategy to prevent metastasis.

## Background

Hepatocellular carcinoma (HCC), a highly heterogeneous malignancy [1], is often diagnosed at an advanced stage with metastasis, at which point the opportunity for surgical intervention has been missed and treatment options are limited [2]. Emerging evidence indicates that many metabolic pathways are reprogrammed in cancer to promote cell proliferation and invasion [3]. In particular, abnormal changes in amino acid metabolism are closely related to the malignant progression of tumors [4]. Notably, the effects of amino acid metabolism reprogramming on tumor metastasis remain unclear. Therefore, systematically exploring the changes in amino acid metabolism and their corresponding underlying mechanisms in HCC metastasis is highly important.

Amino acids are essential substances for protein synthesis and have multiple functions in normal cells. Amino acids not only are key factors in cell growth and repair but also participate in the synthesis of crucial biomolecules such as cell membranes, enzymes, and hormones [5]. Compared with normal cells, tumor cells exhibit higher amino acid requirements and dependence [4]. Recent studies have shown that amino acid metabolism reprogramming can affect the proliferation and invasion of tumor cells [4], including breast cancer [6], triple-negative breast cancer [7], mixed lineage leukemia 1 (MLL1) rearranged leukemia [8], and HCC [9]. Moreover, the role of amino acids in the malignant progression of tumors has gradually been revealed. They not only participate in regulating energy generation [10], macromolecular synthesis [11], and signal transduction [12] but also promote tumor development through binding to proteins. For example, the binding of arginine to RNA-binding motif protein 39 can regulate the expression of metabolic genes, thereby maintaining high arginine levels and promoting oncogenic metabolism [9]. Similarly, the binding of leucine to SAR1B activates the mechanistic target of rapamycin complex 1 signaling pathway, thereby promoting tumor cell proliferation [12]. These findings highlight the critical role of amino acid-binding proteins in tumor progression, indicating that amino acids are important metabolites regulating tumor development.

As an important amino acid, proline has gradually attracted attention for its role in tumors. Studies have shown that proline metabolism is abnormally up-regulated in several tumors, including breast cancer [13], prostate cancer [14], and renal cell carcinoma [15]. The activity of proline metabolic enzymes is also abnormal in liver cancer, where the synthesis process is excessively activated [16]. However, the specific mechanism by which proline drives tumor progression is still unclear, and whether proline participates in the progression of liver cancer through its interaction with proteins is still unknown. Therefore, screening for proline-binding proteins and investigating their mechanisms may yield highly valuable insight. Glutamyl-prolyl-tRNA synthetase 1 (EPRS1) is a key l-proline-binding protein involved in protein synthesis. EPRS1 is a bifunctional tRNA synthetase that catalyzes the attachment of glutamate and proline to cognate transfer RNA for subsequent protein synthesis [17]. Recent studies show that nuclear-localized EPRS1 interacts with poly (adenosine diphosphate-ribose) polymerase 1 (PARP1) to enhance its enzymatic activity, promoting PARP1-mediated DNA repair and accelerating breast cancer progression [18]. Moreover, up-regulation of EPRS1 expression in HCC can promote the proliferation, stemness, and migration of cancer cells [19]. However, the specific mechanism by which l-proline binds to EPRS1 to regulate liver cancer metastasis is still unclear.

In this study, we observed abnormal activation of proline metabolism in HCC, especially in metastatic HCC. Innovatively, we found that l-proline promotes HCC metastasis through binding to EPRS1. Mechanistically, l-proline-bound EPRS1 facilitates HCC metastasis by promoting the translation of proline–proline (PP) motif-containing proteins, including phosphatidylinositol-4,5-bisphosphate 3-kinase catalytic subunit α (PIK3CA), AKT serine/threonine kinase 3 (AKT3), and integrin subunit β1 (ITGB1). More importantly, metastasis of HCC can be effectively inhibited by using bersiporocin, an EPRS1 inhibitor that prevents l-proline binding to EPRS1. In summary, our study not only highlights the critical role of the l-proline–EPRS1 axis in HCC progression but also underscores the therapeutic potential of targeting this pathway for the management of HCC.

## Materials and Methods

### Cell lines and cell culture

The human HCC cell lines MHCC-97H and HCC-LM3 were obtained from the Liver Cancer Institute of Zhongshan Hospital, Fudan University (Shanghai, China). The human hepatoblastoma cell line HepG2 (catalog #HB-8065) was procured from the American Type Culture Collection (ATCC). Primary human hepatocytes (PHHs; catalog #LV-PHH001/2) were acquired from Liver Biotechnology (Shenzhen, China). Mouse H22 HCC cell lines (catalog #CBP60230) were purchased from Cobioer Biosciences (Nanjing, China).

MHCC-97H, HCC-LM3, and HepG2 cells were maintained in Dulbecco’s modified Eagle’s medium (DMEM; catalog #D6429, Sigma-Aldrich) supplemented with 10% fetal bovine serum (FBS; catalog #10270, Gibco) and 1% penicillin–streptomycin (catalog #15140122, Gibco). PHHs were cultured in a dedicated maintenance medium (catalog #LV-WEM001, Liver Biotechnology) containing 1% penicillin–streptomycin. H22 cells were grown in RPMI 1640 medium (catalog #30-2001, ATCC) supplemented with 10% FBS and 1% penicillin–streptomycin.

All cell lines were incubated at 37 °C in a 5% CO₂ atmosphere. The human cell lines were authenticated by short tandem repeat profiling, and all lines were routinely tested to confirm the absence of mycoplasma contamination.

### Orthotopic HCC mouse model

All mice were maintained under specific pathogen-free conditions at an ambient temperature of 20 to 26 °C with a 12-h light/12-h dark cycle. Euthanasia was performed via CO₂ inhalation when mice met the predefined humane endpoints, including ≥20% body weight loss relative to baseline, loss of mobility, and reduced activity. The study protocol was approved by the Animal Ethics Committee of Chongqing Medical University (IACUC-CQMU-2024-0202).

Male BALB/c nude mice were purchased from Byrness Weil Biotech Ltd. To establish an orthotopic HCC mouse model, 1.5 × 10^6^ MHCC-97H cells stably expressing green fluorescent protein (MHCC-97H-GFP) in 50% Matrigel (catalog #356234, Corning) were orthotopically injected into the left lateral lobe of the mouse liver. Mice were randomly assigned to 5 groups (*n* = 6 per group) using a computer-based randomizer. During the 2 to 5 weeks post-implantation, they were euthanized and subjected to fluorescence imaging (IVIS Spectrum, PerkinElmer) to analyze liver tumors and lung tissues. Liver tumors collected at the indicated time points were immediately frozen in liquid nitrogen for quantitative proteomics analysis; mouse serum was subjected to targeted metabolomics, and tissues were embedded for hematoxylin and eosin (H&E) staining.

To assess the role of l-proline in HCC metastasis, we established an orthotopic HCC mouse model by injecting 1.5 × 10^6^ MHCC-97H-GFP cells into the left lateral liver lobe. Using this model, we performed 2 independent studies: (a) In one study, mice received intraperitoneal injections of either phosphate-buffered saline (PBS; *n* = 10) or l-proline (500 mg/kg; catalog #P5607, Sigma-Aldrich; *n* = 10) every 2 d. (b) In a separate study, mice were randomized to receive either a control diet (CD; *n* = 10) or a proline-free diet (NPD; catalog #A23053002, SYSE BIO; *n* = 10). Mice were euthanized 5 weeks after implantation. Metastatic lung tumor nodules and liver tumor nodules were counted using H&E staining and fluorescence imaging.

To investigate the effect of EPRS1 on HCC metastasis, 1.5 × 10^6^ MHCC-97H cells with or without stable *EPRS1* knockout *(sgEPRS1 #1*; *n =* 10 per group) were orthotopically injected into the left lateral lobe of the liver. Mice were euthanized 5 weeks after implantation.

### Patient tissue and serum

This study utilized clinical samples from 2 independent cohorts. Cohort 1 from the First Affiliated Hospital of Chongqing Medical University included serum and tissue samples: (a) Serum samples were collected from 91 HCC patients [including 50 patients with no metastasis (NM) and 41 with extrahepatic metastasis (EHM)] and 20 healthy individuals (control; Table S1). These samples were subjected to amino acid profiling. (b) Paired tumor and adjacent normal tissues were collected from 36 HCC patients (NM, *n* = 18; EHM, *n* = 18; Table S2). Tumor tissues from a subset of 17 patients (NM, *n* = 9; EHM, *n* = 8) selected from the 36 HCC patients described above were subjected to RNA sequencing (RNA-seq; Table S3). The paired tissues of 36 patients were analyzed by Western blotting, proline content determination, quantitative real-time polymerase chain reaction (RT-qPCR), and immunohistochemistry (IHC). Cohort 2, comprising 176 HCC patients (NM, *n* = 116; EHM, *n* = 60) and 52 controls from the Tianjin Medical University Cancer Institute and Hospital, provided serum samples for independent amino acid profiling (Table S4).

All enrolled HCC patients met the following inclusion criteria: (a) tumor, node, metastasis (TNM) stages I to IV according to the American Joint Committee on Cancer (AJCC)/Union for International Cancer Control (UICC) eighth edition system; (b) pathologically confirmed HCC; and (c) for tissue-based analyses, underwent surgical resection. This study was conducted in a blind manner and with approval from the Ethics Committee of Chongqing Medical University (approval number: 2024061) and Tianjin Medical University Cancer Institute and Hospital (approval number: bc20254421), in accordance with the principles of the Declaration of Helsinki. Informed consent was obtained from all participating patients.

### Targeted metabolomics profiling

The targeted metabolomics profiling (including amino acids and their derivatives) of human and mouse serum was carried out by Shanghai Applied Protein Technology Co. Ltd. To extract metabolites from the samples, an appropriate amount of precooled methanol/acetonitrile (1:1, v/v) was added to the samples and adequately vortexed. The samples were then centrifuged at 14,000*g* at 4 °C for 20 min, after which the collected supernatant was dried under vacuum. Before liquid chromatography–tandem mass spectrometry (LC-MS/MS) analysis, the dried metabolite extract was redissolved in 100 μl of an aqueous acetonitrile solution (acetonitrile:water = 1:1, v/v). After thorough vertexing, the mixture was centrifuged at 14,000*g* and 4 °C for 15 min. The resulting supernatant was collected for analysis.

Analyses were performed using an Ultra High Performance Liquid Chromatography system (Agilent 1290 Infinity II) coupled to a QTRAP 6500+ LC-MS/MS system (SCIEX). The analytes were separated on an Amide column (catalog #186004801, Waters) and a C18 column (catalog #186002352, Waters). Metabolites were quantified using MultiQuant (version 3.0.3, SCIEX) or Analyst software (version 1.6, SCIEX).

### Quantitative proteomics analysis

The 4D-FastDIA quantitative proteomic analysis of tumor tissues was carried out by Jingjie PTM Biolabs Inc. (Hangzhou, China). We collected the tumor tissues from the orthotopic HCC mouse models for quantitative proteomics analysis at a series of time points (weeks 2, 3, 4, and 5) since the HCC cells were injected into the mouse liver (*n =* 6 per group). The experiments were performed according to the standard protocols. In brief, samples were immediately frozen in liquid nitrogen and then lysed with lysis buffer (1% Triton X-100, 1% protease inhibitor cocktail) using a high-intensity ultrasonic processor for 3 min on ice. Then, the protein samples were digested with trypsin and reduced and alkylated with 5 mM dithiothreitol (DTT) and 11 mM iodoacetamide, and finally desalted using Strata X SPE columns (catalog #8B-S100-AAK, Phenomenex). Next, the tryptic peptides were dissolved in solvent A (0.1% formic acid, 2% acetonitrile/in water) and solvent B (0.1% formic acid, 90% acetonitrile/in water). Peptide separation was performed using a linear gradient of solvent B, where the percentage indicates the proportion of the strong elution solvent B in the mobile phase. The gradient profile was as follows: 0 to 22.5 min, 6% to 22% B; 22.5 to 26.5 min, 22% to 34% B; 26.5 to 28.5 min, 34% to 80% B; and 28.5 to 30 min, 80% B. This separation was performed on an Easy-nLC 1200 nanoflow ultrahigh-pressure liquid chromatography (UPLC) system (Thermo Fisher Scientific) at a constant flow rate of 700 nl/min. The separated peptides were analyzed in Orbitrap Exploris 480 (Thermo Fisher Scientific) with a nano-electrospray ion source.

Data-independent acquisition (DIA) data were analyzed using DIA-NN software (version 1.8) against the UniProt Homo sapiens reference proteome (Homo_sapiens_9606_SP_20231220.fasta). To control the false discovery rate (FDR), we employed the integrated target-decoy strategy in DIA-NN: First, the software automatically generates a decoy database containing biologically nonexistent sequences that share statistical properties with target sequences, serving as internal references to estimate false matches in the target database [20]; then, DIA-NN calculates the FDR by dividing the number of decoy matches exceeding the score threshold by the number of target matches exceeding the same threshold [21], with the FDR threshold set to <1% at both peptide and protein levels.

### Bioinformatic analysis

To assess the overall structural differences in the distinct amino acid metabolomic features in patients with HCC, we performed principal coordinates analysis (PCoA) using the Vegan R package [22]. Permutational multivariate analysis of variance (PERMANOVA) was used to determine that observed differences in clustering between groups were statistically significant.

To elucidate the dynamic pattern of amino acid metabolites and protein expression over time, we performed clustering analysis of metabolites and proteins using the R package “Mfuzz” [23]. The optimal number of clusters was determined by the elbow method [24,25]. Metabolites were subsequently assigned to specific clusters based on their calculated membership values [26]. For the proteomic data, we first focused on proteins associated with the Reactome pathway “Metabolism of amino acids and derivatives” (R-HSA-71291). These selected proteins underwent the same clustering analysis described above. To interpret the biological functions of the resulting protein clusters, Kyoto Encyclopedia of Genes and Genomes (KEGG) pathway enrichment analysis was performed using the “clusterProfiler” R package [27].

To evaluate the expression of pyrroline-5-carboxylate synthetase (P5CS), pyrroline-5-carboxylate reductase 1 (PYCR1), and catabolic enzyme proline dehydrogenase (PRODH) across multiple HCC datasets, we obtained their expression level data from the online tool Integrative HCC Gene Analysis (IHGA) (https://www.hccdatasph.cn/app/ihga). This platform provides integrated access to transcriptomic data from multiple public HCC datasets (including TCGA-LIHC and GEO series). Data visualization was performed using the OmicShare tool (https://www.omicshare.com/tools). In addition, to assess their relevance to HCC metastasis/relapse, we performed this analysis using the publicly available GSE14520 dataset [28]. Based on the predicted risk metastasis signature defined in that study, we stratified the patients and compared the expression levels of mRNA levels of *P5CS*, *PYCR1*, and *PRODH* between these groups. Data visualization was performed using GraphPad Prism (version 9.5).

To identify metastasis-associated genes, we retrieved the curated metastasis-related gene set from the Human Cancer Metastasis Database (HCMDB) [29] and used it for subsequent analyses.

### RNA-seq analysis of clinical HCC samples

RNA-seq and data analysis were performed by Shanghai Sinotech Genomics Co. Ltd. (Shanghai, China). Tumor tissues for RNA-seq analysis were collected from cohort 1, including 17 HCC patients (NM, *n* = 9; EHM, *n* = 8; Table S3). TRNzol reagent (catalog #DP424, Tiangen) was used for total RNA isolation. Sequencing was then performed using the NovaSeq 6000 (Illumina) instrument to construct a single-end sequencing library.

### Quantitative real-time PCR

Total RNA was extracted from cells or tumor samples using TRNzol reagent, and cDNA was synthesized using an RT Reagent Kit (catalog #KR116, Tiangen), followed by real-time PCR with SYBR Green (catalog #1725121, Bio-Rad) and specific primers (Table S5). The fold changes of target gene expression were determined using the 2^−∆∆Ct^ method. β-Actin was used as an internal control.

### Pull-down assay and LC-MS/MS analysis

Biotin-labeled l-proline (bio-l-proline) was custom-synthesized by TGTMED Biotechnologies (Shanghai, China). To detect l-proline-binding proteins, 300 μl of bio-l-proline (5 mM) was added to a spin column and incubated at 4 °C. Then, 250 μl of the biotin blocking solution was added to the rotary column and incubated for 5 min at room temperature. Subsequently, protein lysates (1 mg) from MHCC-97H or HCC-LM3 cells were loaded onto a rotating column and incubated at 4 °C. Finally, the beads were washed and collected.

The proteins were reduced and alkylated with DTT and iodoacetamide, followed by digestion with trypsin. The dried peptides were desalted by Monospin Desalting Column (catalog #5010-21701, GL Sciences) and analyzed by EASY-nLC 1000 nano-liquid chromatography system coupled with an Orbitrap Fusion Lumos mass spectrometer (Thermo Fisher Scientific). The vacuum-dried samples were redissolved with 0.1% formic acid and separated on a custom-packed C18 column (75 μm inner diameter × 25 cm, 1.9 μm beads, 120 Å pores). The flow rate was 300 nl/min. Data were acquired by full scan [mass/charge ratio (*m*/*z*) 350 to 1,800, 60,000 resolution]. At a normalized collision energy of 30%, the precursor ions are fragmented in high-energy collision dissociation. The raw files were analyzed by Proteome Discoverer 2.4 (version 2.4, Thermo Fisher Scientific) using the SwissProt database. The fixed modifications included carbamidomethyl (C), and the variable modifications included oxidation (M) and acetyl (N-Term).

To verify the interaction, bio-l-proline or custom-synthesized biotin-labeled glutamate (Wuhan Haode Peptide Co. Ltd.) was added to 50 μl of streptavidin magnetic beads (catalog #88817, Thermo Fisher Scientific) and incubated for 30 min at 4 °C. d-Biotin was used as a control. Then, the lysates prepared from MHCC-97H and HCC-LM3 cells were added to streptavidin magnetic beads and incubated for 24 h at 4 °C with gentle rotation. The samples were then washed 3 times for Western blotting analysis.

### Surface plasmon resonance analysis

The binding of l-proline with EPRS1 was examined using surface plasmon resonance (SPR) on a BIAcore X100 system. First, 10 μg/ml of EPRS1 or prolyl-tRNA synthetase 1 (PARS1) was immobilized on a CM5 chip according to the protocol of the Amine coupling kit (catalog #BR-1000-50, Cytiva). Next, l-proline or d-proline was prepared in PBS (catalog #B640435, Sangon Biotech) with 0.05% Tween 20 (catalog #P1379, Sigma-Aldrich). The binding of l-proline to EPRS1 or PARS1 was carried out at 25 °C with an influx of 30 μl/min through the chip channel, utilizing a concentration gradient of l-proline ranging from 62.5 to 1,000.0 μM. The binding kinetics, including the association rate constant (*K*_a_), dissociation rate constant (*K*_d_), and equilibrium dissociation constant (*K*_D_), were analyzed and calculated using Biacore X100 evaluation software (version 2.0.1, GE HealthCare).

### Computational docking and molecular simulation

Molecular docking of the EPRS1 protein or EPRS1^E1123A^ and the small-molecule l-proline was performed by AutoDock Vina (version 1.2.3, Scripps Research Institute). The Gromacs software (version 2022.3) was used for molecular dynamics simulation, and the built-in tool of the software was used to analyze the trajectory, root mean square deviation (RMSD), hydrogen bonding, Gibbs energy landscape, radius of gyration (Rg), and the free energy contribution. Finally, based on the free energy of each residue, the key residues involved in small-molecule ligand interactions were identified.

### Western blotting

Cell or tumor samples were lysed in radioimmunoprecipitation assay (RIPA) buffer containing protease inhibitor cocktail (catalog #04693132001, Roche) for total protein extraction. Protein concentration was quantified using the bicinchoninic acid assay kit (catalog #23227, Thermo Fisher Scientific). Then, the protein was separated by sodium dodecyl sulfate–polyacrylamide gel electrophoresis (SDS-PAGE) and transferred to polyvinylidene fluoride film (catalog #10600023, GE HealthCare). After blocking with 5% nonfat milk, membranes were incubated with the indicated primary antibody at 4 °C overnight. After washing, the membranes were incubated with the corresponding secondary antibody, and the signal was visualized using enhanced chemiluminescence (ECL) Western blotting reagent (catalog #WBKLS0500, Millipore). Glyceraldehyde-3-phosphate dehydrogenase (GAPDH) was used as a loading control; the antibodies used are shown in Table S6. The intensity of protein bands was analyzed with ImageJ.

### Immunohistochemistry

HCC tissue samples for IHC were collected from cohort 1 (Table S2). Tissue samples were fixed in formalin for 24 to 48 h and then embedded in paraffin and sectioned into 4-μm slices. After drying at 95 °C for 10 min, the sections were deparaffinized in xylene, rehydrated through a graded ethanol series, and subjected to microwave-assisted antigen retrieval in 10 mM sodium citrate buffer (pH 6.0) at 95 to 100 °C for 20 min. Subsequently, endogenous peroxidase activity was blocked with endogenous peroxidase blocking buffer (catalog #P0100A, Beyotime), and nonspecific binding sites were blocked with 10% normal goat serum (catalog #ZLI-9056, ZSGB-Bio). The sections were then incubated with primary antibodies (Table S6) overnight at 4 °C, followed by incubation with a horseradish peroxidase (HRP)-conjugated secondary antibody (catalog #KIT9902, Maixin Biotech) for 1 h at room temperature. Immunoreactivity was visualized using diaminobenzidine (DAB; catalog #ZLI-9018, ZSGB-Bio) as the chromogen, and the nuclei were counterstained with hematoxylin. Slides were mounted with neutral balsam (catalog #G8590, Solarbio). Positive controls (tissue known to express the target antigen) and negative controls (omission of the primary antibody) were included in each staining batch to ensure specificity and validate the protocol. Finally, all slides were imaged under a microscope, and the IHC staining score was calculated using ImageJ software based on both the staining area and intensity. The staining area was graded as follows: 0 (0% to 5%), 1 (5% to 25%), 2 (25% to 50%), 3 (50% to 75%), and 4 (>75%). The staining intensity was graded as: 0 (negative), 1 (weak), 2 (moderate), and 3 (strong). A final IHC score (ranging from 0 to 12) for each sample was generated by multiplying the area score by the intensity score.

### Proline content determination

Proline was determined using a proline content determination kit (catalog #BC0290, Solarbio) following the manufacturer’s instructions. Briefly, the extraction solution was added to samples comprising cells, tumor tissues from mouse models, and paired tumor/adjacent normal tissues from cohort 1 HCC patients (NM, *n* = 18; EHM, *n* = 18). Cells were lysed by sonication, and tumor tissues were homogenized on ice. The resulting supernatant was shaken and incubated in boiling water for 10 min. After centrifugation at 10,000*g* for 10 min at room temperature, the supernatant was collected. It was then mixed with reagent 1 and reagent 2 (provided in the proline content determination kit), followed by incubation in a boiling water bath for 30 min. After cooling, the absorbance was measured at 520 nm using a microplate reader (BioTek Synergy H1 Multimode Reader, Agilent).

### Multiplex immunofluorescence staining

Multiplex immunofluorescence was performed on formalin-fixed, paraffin-embedded HCC tissue sections using a multiplex fluorescence immunohistochemistry kit (catalog #10079100020, Panovue), according to the manufacturer’s instructions. In brief, sections were deparaffinized, hydrated, antigen-repaired, and blocked in 3% bovine serum albumin (BSA) (catalog #9048-46-8, Sangon Biotech) at room temperature for 1 h, and then incubated with the corresponding primary antibody overnight at 4 °C. Signal amplification was performed after incubation with the secondary antibody, and nuclei were counterstained with 4′,6-diamidino-2-phenylindole (DAPI; catalog #62248, Thermo Fisher Scientific) after the completion of all indicator staining. The antibodies used are shown in Table S6. The images were captured with a confocal microscope (TCS SP8, Leica).

### Immunofluorescence

Coverslips seeded with cells were fixed with paraformaldehyde, permeabilized with 0.5% Triton X-100 solution, and blocked with 3% BSA. The coverslips were then incubated with the corresponding primary antibodies overnight at 4 °C. The next day, sections were incubated with immunofluorescent secondary antibodies for 2 h at room temperature. After washing 3 times, the nuclei were counterstained with DAPI. The antibodies used are shown in Table S6. The images were captured with a confocal microscope (TCS SP8, Leica).

To determine the colocalization of bio-l-proline or d-biotin with EPRS1, the cells were pretreated with biotin or bio-l-proline (1 mM) for 2 h and then fixed and incubated with EPRS1 antibodies as described previously. The next day, sections were incubated with Alexa Fluor 555-conjugated goat anti-mouse immunoglobulin G (IgG) (catalog #ab150078, Abcam) and avidin–Alexa Fluor 488 (catalog #ab150077, Abcam) at a dilution of 1:1,000 for 2 h at room temperature. After washing 3 times, the nuclei were counterstained with DAPI. The images were captured with a confocal microscope (TCS SP8, Leica).

### Lentivirus transduction and cell line construction

To construct *EPRS1*-knockout and *P5CS*-knockdown HCC cell lines, the *sgEPRS1* and *shP5CS* lentivirus purchased from Genchem Co. Ltd. (Shanghai, China) were used to infect HCC cell lines. After 14 d of treatment with 2 μg/ml puromycin, the stable knockout or knockdown clones were serially diluted to obtain single-cell clones.

### Invasion and migration assay

For the wound-healing assay, cells were treated with 5 μg/ml mitomycin C (catalog #A11491, AdooQ Bioscience) for 2 h, after which a pipette was used to produce monolayer wounds. Images were captured with Incucyte Live-Cell Analysis System (Sartorius) at the indicated times after washing with PBS. The migration rate was measured using ImageJ and defined as the percentage of the initial wound width that was covered by HCC cells after 48 h, using the following formula: Migration rate (%) = [(*W*₀ − *W*_1_)/*W*₀] × 100%. *W*₀ represented the initial wound width at 0 h, and *W*_1_ represented the wound width at 48 h.

For the Transwell assays, the cells were cultured in the presence of 5 μg/ml mitomycin C for 2 h, and then 1 × 10^5^ HCC cells were resuspended in serum-free medium and seeded into the upper chamber. For the migration assay, cells were seeded into uncoated inserts (catalog #353097, FALCON). For the invasion assay, cells were seeded into inserts precoated with Matrigel (catalog #354480, Corning). Serum-containing medium was added to the lower chamber. After incubation at 37 °C in a 5% CO_2_ incubator, Transwell membranes were fixed with 4% formaldehyde and stained with 0.1% crystal violet (catalog #V5265, Sigma-Aldrich). The number of migrated or invaded cells was counted in the 6 randomly selected fields under an inverted microscope.

For the rescue experiment with metabolite supplementation, *P5CS*-knockdown HCC cells were seeded and supplemented with l-proline (1 mM), glutamine (4 mM), NADPH [reduced form of nicotinamide adenine dinucleotide phosphate] (1 mM), or collagen I (0.2 μg/ml) for 24 h. Following the supplementation treatments, the migratory and invasive capabilities of the cells were immediately assessed using the Transwell assays as described above. All reagents, including catalog numbers, are listed in Table S6.

### Three-dimensional spheroid invasion assay

Invasion assays were performed using Cultrex 3D Spheroid Basement Membrane Extract Cell Invasion Assay (catalog #3500-096-K, R&D Systems). According to the manufacturer’s instructions, 4,000 cells were mixed with 50 μl of 1× spheroid formation extracellular matrix (ECM) and added to a well spheroid formation plate. After 2 to 3 d of incubation, the invasion matrix and medium containing 20% FBS were added. Images were captured at 0 and 48 h post-embedding in the invasion matrix. The extent of invasion was quantified using the following formula: Invasion area = *I*_1 −_ *I*_0_. *I*_0_ represented the initial spheroid area at 0 h, and *I*_1_ represented the spheroid area at 48 h.

### F-actin staining

HCC cells with the indicated treatments were seeded on coverslips in 12-well plates and cultured for 24 h. The cells were washed twice with PBS, then fixed with 4% paraformaldehyde, and finally stained with DyLight 488 phalloidin (catalog #12935S, Cell Signaling Technology) and DAPI. The images were captured with a confocal microscope TCS SP8 (Leica).

### Protein synthesis and degradation assays

For the puromycin incorporation assay, HCC cells with the indicated treatments were treated with puromycin (1 μM) at 37 °C for 30 min, after which the new protein synthesis rate was measured using anti-puromycin antibodies (catalog #MABE343, Merck).

The Click-iT protein synthesis was measured using the Click-&-Go Plus OPP Protein Synthesis Assay Kit (catalog #CCT-1493, Vector Laboratories), according to the manufacturer’s instructions. HCC cells with the indicated treatments were treated with 20 μM o-propargyl-puromycin (OPP; provided in the Click-&-Go Plus OPP Protein Synthesis Assay Kit) for 30 min. After washing in PBS, cells were stained in a reaction cocktail containing AZDye 488 Azide (provided in the Click-&-Go Plus OPP Protein Synthesis Assay Kit) for 20 min in the dark. The images were captured with a confocal microscope (TCS SP8, Leica).

To measure protein synthesis in vivo, tumor cells were extracted from the tumor tissues using the Tumor Enzymatic Digestion Kit (catalog #DHTE-5001, RWD Life Science). Briefly, tumor tissues were cut and mixed with the enzymatic digestion buffer according to the manufacturer’s instructions. The digested tissues were transferred to tissue treatment tubes for single-cell suspension preparation. Digestion was stopped by adding culture medium, and the cell suspension was collected through a cell filter. The cells were treated with 20 μM OPP for 30 min. Subsequently, Click-iT Plus Alexa Fluor 647 Picolyl Azide Toolkit (catalog #C10643, Thermo Fisher Scientific) was used for the click reaction. All samples were then analyzed by flow cytometry (CytoFLEX, Beckman), and data were processed using FlowJo software (version 10, BD Biosciences).

To assess whether the reduced protein expression was due to proteasomal degradation, HCC cells were treated with l-proline (1 mM, 24 h) or with the combination of l-proline and bersiporocin (10 μM, 24 h; catalog #T39739, TargetMol), followed by treatment with or without 10 μM MG132 (catalog #M7449, Sigma-Aldrich) for 12 h. Cells were subsequently harvested, and the protein expression levels of PIK3CA, AKT3, and ITGB1 were analyzed by Western blotting.

### Ribosome profiling and paired RNA-seq in cell models

The ribosome profiling (Ribo-seq) and RNA-seq libraries were sequenced on the Illumina sequencing platform by Genedenovo Biotechnology Co. Ltd. (Guangzhou, China). MHCC-97H cells were treated with or without bersiporocin (10 μM) for 24 h and then collected for Ribo-seq. The cells were incubated with medium containing 100 μg/ml cycloheximide (CHX) for 15 min. After discarding the medium, the cells were washed twice with PBS prechilled to 4 °C and containing CHX, and the samples were immediately frozen in liquid nitrogen.

For ribosome footprint (RF) preparation, ribonuclease (RNase) I (catalog #M0307, NEB) and deoxyribonuclease (DNase) I (catalog #M0303, NEB) were added to 400 μl of lysate and then gently mixed on a nutator mixer at room temperature and incubated for 45 min. Nuclease digestion was stopped by adding 10 μl of SUPERase·In RNase inhibitor (catalog #AM2696, Invitrogen). The size exclusion column (catalog #27-5140-01, GE HealthCare) was equilibrated by gravity flow with 3 ml of polysome buffer and then centrifuged at 600*g* for 4 min at room temperature. To isolate RFs larger than 17 nucleotides, 100 μl of digested RFs was passed through a column by centrifugation at 600*g* for 2 min and eluted with SDS. Subsequently, short (50 to 80 bases) antisense DNA probes complementary to rRNA were added to the RF solution, followed by digestion with RNase H (catalog #H0110, NEB) and DNase I to remove rRNA and residual probes. Finally, RFs were further purified using magnetic beads.

For library construction and sequencing, adapters were added to both ends of the RFs, followed by reverse transcription and PCR amplification. PCR products of 140 to 160 base pairs were enriched to construct cDNA libraries, which were subjected to Illumina HiSeq X10 sequencing by Genedenovo Biotechnology Co. Ltd. (Guangzhou, China).

For RNA-seq, total RNA was extracted using TRNzol reagent. The extracted mRNA was enriched using mRNA capture beads. After purification, the mRNA was fragmented at 80 °C. The fragmented mRNA was then used as a template to synthesize the first strand of cDNA in a reverse transcriptase mixture system. During the synthesis of the second strand of cDNA, end repair and A-tailing are completed. Next, adapters were ligated to the ends of the cDNA fragments. The ligation product was then purified and size-selected using Hieff NGS DNA selection beads (catalog #12601ES, Yeasen Biotechnology) to enrich for fragments of the desired length. PCR library amplification was then performed using NovaSeq X Plus (Illumina).

### Polysome profiling

MHCC-97H cells were treated with l-proline (1 mM; 24 h), bersiporocin (10 μM; 24 h), or their combination. Then, all groups were treated with 100 μg/ml CHX for 15 min to freeze ribosomes on mRNAs in the elongation phase and then lysed on ice, and the supernatant was added to a sucrose gradient. The gradients were centrifuged at 4 °C for 5 h at 115,460*g*. After classifying the fractions, RNA was extracted using the TRNzol reagent, and the levels of mRNA were detected by RT-qPCR. The primer sequences used are listed in Table S5.

### Luciferase reporter assay

The sequences of *PIK3CA*, *AKT3*, and *ITGB1* were cloned into the dual luciferase vector pmirGLO (catalog #E1330, Promega), and the plasmid was synthesized by Youbao Biotechnology (Chongqing, China). HCC cells were transfected with the plasmid for 24 h, and luciferase activity was measured using a dual-luciferase reporter assay system (catalog #E1910, Promega) in accordance with the manufacturer’s instructions. The Renilla luciferase gene was employed as a reference reporter. The mRNA abundance of firefly luciferase (F-luc) and renilla luciferase (R-luc) was measured by RT-qPCR, and the translation efficiency (TE) was calculated as the reporter protein yield (F-luc/R-luc) divided by mRNA abundance.

### Patient-derived orthotopic xenograft model

Patient-derived orthotopic xenograft (PDOX) models were purchased from BEIJING IDMO Co. Ltd. (Beijing, China). The tissues from subcutaneously seeded NM and EHM tumors were orthotopically implanted into the left lateral liver lobe of NOD/ShiLtJGpt-Prkdc^em26Cd52^Il2rg^em26Cd22^/Gpt (NCG) mice. Clinical information of the corresponding donor patients is provided in Table S7. The liver tumor volume was subsequently measured, and the metastatic lung nodules were counted.

To evaluate the in vivo efficacy of the combination therapies, mice bearing EHM-derived tumors were randomly assigned to 4 groups (*n* = 5 per group): (a) vehicle control, (b) bersiporocin alone, (c) bersiporocin combined with alpelisib, and (d) bersiporocin combined with MK-2206. Bersiporocin was administered at 10 mg/kg by intraperitoneal injection every 2 d. Alpelisib (catalog #T1921, TargetMol) was administered at 25 mg/kg by oral gavage daily, and MK-2206 (catalog #T1952, TargetMol) was administered at 100 mg/kg by intraperitoneal injection once per week. All treatments were initiated 1 week after tumor implantation and continued for 4 weeks. The liver tumor volume was subsequently measured, and the metastatic lung nodules were counted.

### Detections of mitochondrial complex activity, adenosine triphosphate, reactive oxygen species, and glutamine levels

The activities of mitochondrial complexes I and IV were measured using the corresponding assay kits (catalog #E-BC-K834-M and catalog #E-BC-K837-M, Elabscience), according to the manufacturer’s instructions.

The cellular levels of adenosine triphosphate (ATP), reactive oxygen species (ROS), and glutamine were measured using the ATP assay kit (catalog #S0026, Beyotime), the ROS assay kit (catalog #CA1420, Solarbio), and the glutamine colorimetric assay kit (catalog #E-BC-K853-M, Elabscience), respectively, according to the manufacturer’s instructions.

### Detection of serum aminotransferase activity

The mice’s blood samples were collected and centrifuged at 3,000*g* for 15 min at 4 °C to obtain serum. The alanine aminotransferase (ALT) levels were measured using an ALT assay kit (catalog #BC1555, Solarbio) according to the manufacturer’s instructions.

### AKT serine/threonine kinase 1/neuroblastoma Ras viral oncogene homolog-induced HCC mouse model

AKT/neuroblastoma Ras viral oncogene homolog (NRAS)-induced HCC mouse model using hydrodynamic transfection was constructed by LiLai Biotechnology Co. Ltd. (Chengdu, China). Briefly, a plasmid mixture consisting of pT3-mAKT1^mut^-EGFP (0.7 μg; custom-synthesized by Chengdu OriginTree Biotechnology Co. Ltd.), pT-mNRAS^3muts^-mCherry (0.7 μg; custom-synthesized by Chengdu OriginTree Biotechnology Co. Ltd.), pT2-shP53 (1.8 μg; catalog #124261, Addgene), and pCMV (CAT)T7-SB100 (0.05 μg; catalog #34879, Addgene) was diluted in 1 ml of sterile physiological saline (0.9% NaCl) and injected into the lateral tail vein of C57BL/6J mouse within 5 to 7 s. Using this model, we performed 2 independent studies: (a) Mice received intraperitoneal injections of either PBS (*n* = 10) or l-proline (500 mg/kg; *n* = 10) every 2 d. (b) Mice were randomized to receive CD (*n* = 10) or NPD (*n* = 10). After receiving the corresponding experimental treatment for 4 weeks, the mice were euthanized.

### H22 spleen injection liver metastasis model

The H22 spleen injection liver metastasis model was established through the intrasplenic injection of H22 cells (5 × 10^5^ cells). Briefly, the mice were anesthetized, and a left subcostal incision was introduced to expose the spleen. H22 cells in 40 μl of PBS were injected into the spleen. The spleen was ligated and removed. Using this model, we performed 2 independent studies: (a) Tumor-bearing mice received intraperitoneal injections of either PBS (*n* = 6) or l-proline (500 mg/kg; *n* = 6) every 2 d. (b) Mice were randomized to receive either CD (*n* = 6) or NPD (*n* = 6). After receiving the corresponding experimental treatment for 2 weeks, the mice were euthanized.

### Plasmid transfection

For expression of recombinant EPRS1 fragments, including Flag-EPRS1, Flag-EPRS1^ΔPARS1^, Flag-EPRS1^ΔEARS1^, Flag-EPRS1^E1123A^, and Flag-EPRS1^E205G^ were synthesized by Youbao Biotechnology (Chongqing, China). HCC cells were seeded and transfected using the Lipofectamine 3000 kit (catalog #L3000015, Invitrogen) according to the manufacturer’s instructions. Cells were harvested 48 h post-transfection and subjected to subsequent functional assays.

### Pharmacokinetic study

The pharmacokinetic study was performed in C57BL/6J mice (*n* = 3 per group). Mice received a single intraperitoneal injection of bersiporocin (10 mg/kg). Plasma samples (15 μl) were collected, and the plasma concentrations of bersiporocin were quantified by a validated LC-MS/MS (TQ5500, SCIEX).

### Statistical analysis

Data presentation was conducted using GraphPad Prism (version 9.5, GraphPad Software Inc.). Differences between 2 groups were assessed by Student’s *t* test, while comparisons involving multiple groups were conducted using one-way analysis of variance (ANOVA) or 2-way ANOVA followed by appropriate post hoc tests, such as Tukey’s or Dunnett. Regarding variables that did not follow a normal distribution, the nonparametric *t* test and Mann–Whitney *U* test was used for comparisons between 2 groups, while the Kruskal–Wallis test was used for multiple-group comparisons. Chi-square test and Fisher’s exact test were employed to analyze categorical data. Overall survival (OS) analysis was performed in the TCGA-LIHC dataset using the GEPIA3 database [30]; disease-free survival (DFS) analysis was conducted on the GSE14520 dataset and analyzed using Kaplan–Meier curves, with comparisons performed by the log-rank test. Correlation analyses were conducted using Spearman’s rank correlation analysis. Statistical significance was set at *P* < 0.05. Data are presented as mean ± standard deviation (SD) unless otherwise stated.

## Results

### Proline metabolic reprogramming is related to HCC metastasis

**Fig. 1.**
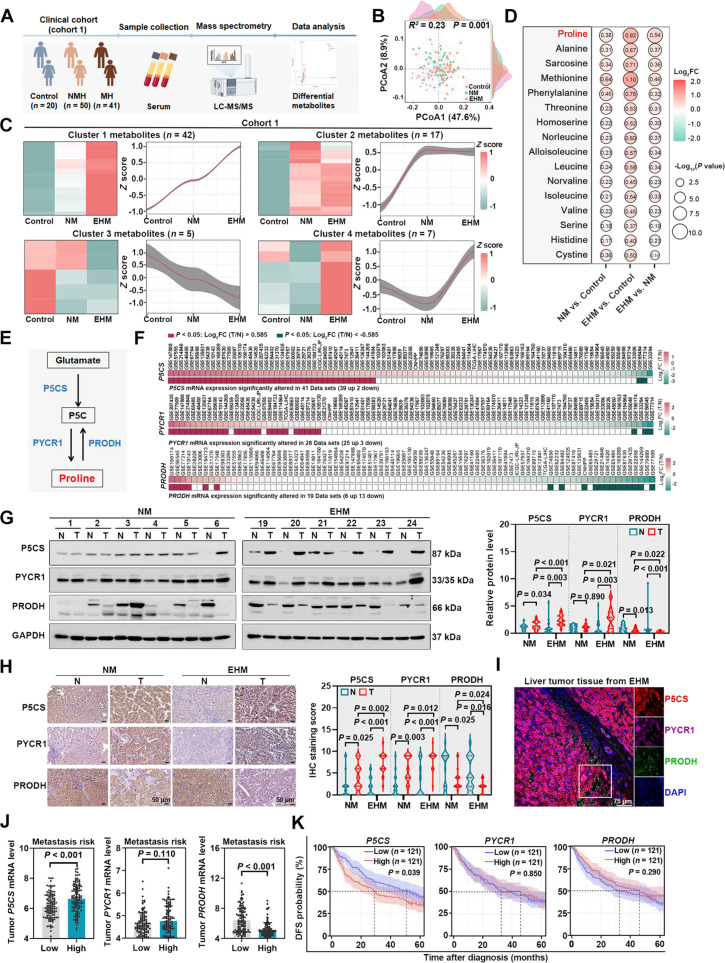
Proline metabolic reprogramming is positively correlated with HCC metastasis. (A) Schematic workflow of metabolomic analysis using serum samples (cohort1, *n* = 111) from healthy individuals (control, *n* = 20), NM (*n* = 50), and EHM (*n* = 41) to identify metastasis-associated amino acids. (B) PCoA of serum amino acid profiles between control, NM, and EHM groups in cohort 1. Statistical significance between groups was determined using PERMANOVA; *R* ^2^ represents the proportion of total variance explained by the grouping factor. (C) Serum amino acid metabolites in cohort 1, identified by LC-MS/MS, were clustered based on their *z*-score normalized expression profiles across the control, NM, and EHM groups. The number in parentheses indicates the metabolite count for each cluster, and the curves depict the mean *z*-score trajectory of each cluster across the groups. The gray shadows around the curves represent the 95% confidence intervals of the curves. (D) Bubble plot showing the expression patterns of 16 serum amino acid metabolites in cohort 1. These amino acid metabolites were identified based on shared consistent up-regulation trends in both the orthotopic HCC mouse model (from week 2 to week 5 post-implantation) and cohort 1 (from control to NM to EHM group). The numbers inside the circle represents Log_2_FC of the metabolite. (E) Schematic showing the key enzymes in proline metabolism: The enzymes P5CS and PYCR1 catalyze proline synthesis, while PRODH catalyzes its degradation. (F) The expression profiles of *P5CS*, *PYCR1*, and *PRODH* were systematically compared between HCC tumor and non-HCC tissues by analyzing multiple public gene expression datasets via the IHGA tool. The boxes present the log_2_FC values in the corresponding datasets. (G) Left: Representative images of Western blotting showing the protein levels of P5CS, PYCR1, and PRODH in paired tumor and adjacent normal tissues from 36 HCC patients in cohort 1 (NM, *n* = 18; EHM, *n* = 18). GAPDH was used as a loading control. The quantification data are shown on the right. (H) Left: Representative IHC images of P5CS, PYCR1, and PRODH protein expression in paired tumor and adjacent normal tissues from HCC patients in cohort 1 (*n* = 36). The quantification data are shown on the right (*n* = 36). (I) Representative multiplexed immunofluorescence images of P5CS (red), PYCR1 (purple), PRODH (green), and DAPI (blue, nuclei) expression in liver tumor tissues from EHM patient (*n* = 3). (J) Analysis of *P5CS*, *PYCR1*, and *PRODH* expression in GSE14520 (*n* = 242), using the predicted risk metastasis signature to stratify patients into high- and low-risk groups (*n* = 121 per group). (K) DFS analysis based on *P5CS*, *PYCR1*, and *PRODH* gene expression levels in HCC patients (GSE14520), with the median expression level serving as the cutoff. Data are presented as mean ± SD (J). *P* values were calculated using a nonparametric *t* test (G and H) and Mann–Whitney *U* test (J). DAPI, 4′,6-diamidino-2-phenylindole; DFS, disease-free survival; EHM, extrahepatic metastasis; FC, fold change; GAPDH, glyceraldehyde-3-phosphate dehydrogenase; IHC, immunohistochemistry; IHGA, integrative HCC gene analysis; LC-MS/MS, liquid chromatography–tandem mass spectrometry; N, adjacent nontumor liver tissue; NM, no metastasis; P5CS, pyrroline-5-carboxylate synthase; PCoA, principal coordinates analysis; PRODH, proline dehydrogenase; PYCR1, pyrroline-5-carboxylate reductase 1; SD, standard deviation; T, primary HCC tissues.

To identify amino acid metabolic alterations during HCC metastasis, we first established the clinical cohort 1, which consisted of 20 healthy individuals (control) and 91 HCC patients (NM, *n* = 50; EHM, *n* = 41; Fig. 1A and Table S1). Serum samples from clinical cohort 1 were collected and subjected to targeted amino acid metabolomics, and a total of 71 amino acid metabolites were identified. As expected, PCoA revealed distinct amino acid metabolomic features in patients with HCC, with separation from controls (Fig. 1B). Based on the elbow method, 71 amino acid metabolites from clinical cohort 1 were clustered into 4 distinct clusters. Notably, among them, 42 amino acid metabolites in cluster 1 showed a progressive increase in levels from control to NM and subsequently to EHM, suggesting a potential relationship with the progression of HCC metastasis (Fig. 1C). To explore the amino acid metabolic alterations during HCC metastasis in vivo, we further established an orthotopic HCC mouse model. Mouse serum samples collected from weeks 2 to 5 post-implantation were subjected to targeted amino acid metabolomics (Fig. S1A and B). In the orthotopic HCC mouse model, 72 serum amino acid metabolites were identified and classified into 6 clusters. Notably, 27 metabolites in cluster 3 exhibited a progressive increase in levels from week 2 to week 5 post-implantation (Fig. S1C). To identify amino acids consistently associated with metastasis across species, we intersected the 42 progressively increasing metabolites in the clinical cohort with the 27 progressively increasing metabolites from the orthotopic HCC mouse model. This analysis yielded 16 amino acids that progressively increased during metastasis in both humans and mice (Fig. 1D and Fig. S1D).

Moreover, we confirmed our finding in an independent validation clinical cohort 2, which consisted of 52 healthy individuals (control) and 176 HCC patients (NM, *n* = 116; EHM, *n* = 60; Table S4). Serum levels of 20 amino acids were analyzed and grouped into 3 clusters according to their expression patterns. The results showed that 12 serum amino acids in cluster 1 (from clinical cohort 2) exhibited a progressive increase in their levels during HCC progression (Fig. S1E). More importantly, by intersecting the progressively increasing serum amino acid profiles across 2 clinical cohorts and orthotopic HCC mouse model, we identified 7 amino acids (proline, serine, alanine, histidine, threonine, methionine, and isoleucine), which shared consistent up-regulation trends and may be relevant to HCC metastasis (Fig. S1F). Functional experiments demonstrated that only exogenous proline supplementation effectively rescued the inhibition of metastasis-related phenotypes caused by amino acid deficiency, which is consistent with the clinical observation that proline levels were up-regulated in metastatic HCC tissues (Fig. S1G and H), suggesting a specific role for proline in HCC metastasis. Meanwhile, we performed the proteomic analysis in tumor tissues from the orthotopic HCC mouse model. From the proteins identified, we selected those associated with amino acid metabolism (Reactome pathway: R-HSA-71291) and then analyzed their expression dynamics. Notably, among the resulting clusters, a total of 55 gradually elevated proteins were identified in cluster 4 (from mouse tumor tissue), which were enriched in the arginine and proline metabolism pathway (Fig. S1I). Taken together, these findings suggest that proline plays an important role during HCC metastasis.

To further elucidate the relationship between proline metabolic reprogramming and HCC metastasis, we systematically quantified the expression of key enzymes governing proline metabolism [16] (Fig. 1E), including anabolic enzymes P5CS and PYCR1 and catabolic enzyme PRODH. We analyzed published datasets using IHGA, which demonstrated increased expression of *P5CS* and *PYCR1* and decreased expression of *PRODH* in HCC tissues compared to non-HCC tissues (Fig. 1F). Moreover, based on the clinical samples from HCC patients, we found that both the protein and mRNA levels of P5CS and PYCR1 were up-regulated in EHM patients compared to NM patients, whereas PRODH was down-regulated (Fig. 1G and Fig. S2A and B), which was further confirmed by IHC staining and multiplexed immunofluorescence staining (Fig. 1H and I). Additionally, aberrant expression of *P5CS* and *PRODH* was associated with a higher risk of metastasis and relapse in HCC (Fig. 1J). However, only elevated *P5CS* expression showed a correlation with poorer DFS and OS, whereas *PYCR1* and *PRODH* showed no such association with prognosis (Fig. 1K and Fig. S2C). Given that P5CS is involved in multiple cancer metabolic processes, including glutamine synthesis [31] and mitochondrial function [32], its specific role in HCC cells remained unclear. Our experiments demonstrated that *P5CS* knockdown reduced intracellular proline levels, while leaving glutamine and ROS levels largely unchanged (Fig. S2D). Since proline metabolism is known to regulate redox balance [33] and collagen biosynthesis [34], we further explored whether the impaired migration and invasion caused by *P5CS* depletion were mediated by specific metabolites or downstream products. We performed rescue experiments by supplementing l-proline, collagen I, NADPH, or glutamine in *P5CS*-knockdown cells. Only proline supplementation was found to restore these phenotypes (Fig. S2E). Consistently, correlation analysis revealed only weak or nonsignificant associations between proline metabolic pathways and redox homeostasis or collagen biosynthesis (Fig. S2F). Taken together, these data suggested that P5CS-mediated proline metabolic reprogramming promoted the metastasis-related phenotypes in HCC.

### l-Proline promotes HCC metastasis

**Fig. 2.**
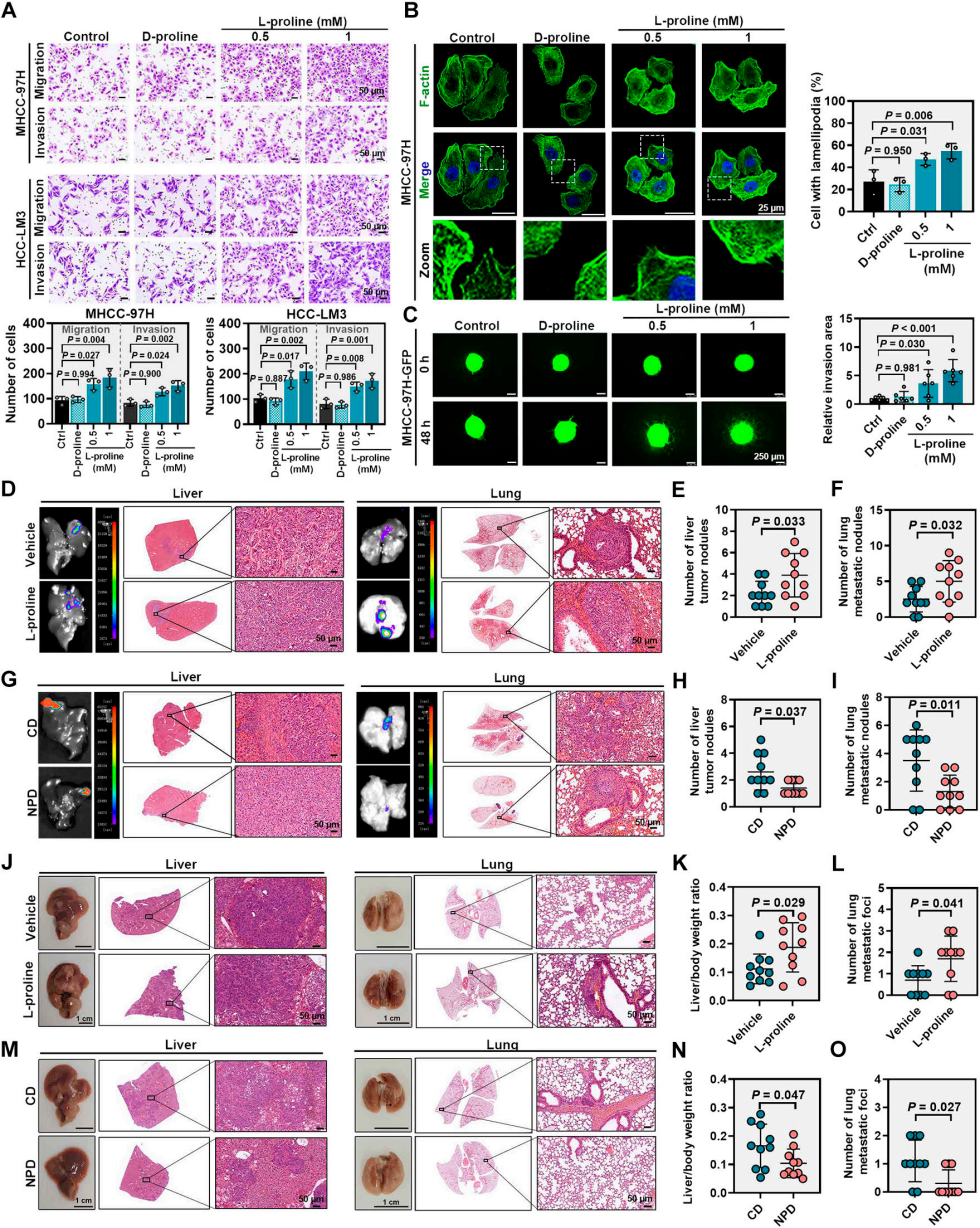
l-Proline promotes HCC metastasis*.* (A) Transwell assays showing the migratory and invasive abilities of MHCC-97H and HCC-LM3 cells treated for 24 h with control (PBS), d-proline (1 mM; negative control), and l-proline (0.5 or 1 mM). The quantification data are shown on the lower panel (*n* = 3 per group). (B) Left: Representative immunofluorescence images of F-actin organization from the indicated treatment conditions in (A). MHCC-97H cells were stained with phalloidin (green, F-actin) and DAPI (blue, nuclei). The quantification data are shown on the right (*n* = 3 per group). (C) Left: Representative images showing the in vitro 3D invasion of MHCC-97H-GFP cells from the indicated treatment conditions in (A). The quantification data were shown on the right (*n* = 6 per group). Images were captured at 0 and 48 h after the spheroids were embedded into the invasion matrix. (D) MHCC-97H-GFP cells were orthotopically injected into the BALB/c nude mice to establish the orthotopic HCC mouse model. Mice were then treated intraperitoneally every 2 d with either vehicle (PBS) or l-proline (500 mg/kg) for 5 weeks (*n* = 10 per group). Representative fluorescence images of liver and lung and corresponding H&E staining were shown. (E and F) Dot plots showing the tumor nodules of liver (E) and lung (F) from the indicated treatment groups in (D). (G) MHCC-97H-GFP cells were orthotopically injected into BALB/c nude mice to establish the orthotopic HCC mouse model. Mice were then fed with either CD or NPD (*n* = 10 per group) for 5 weeks. Representative fluorescence images of liver and lung, and corresponding H&E staining, were shown. (H and I) Dot plots showing the nodules of liver (H) and lung (I) from the indicated treatment groups in (G). (J) AKT/NRAS-induced HCC mouse models were treated intraperitoneally every 2 d with either vehicle (PBS) or l-proline (500 mg/kg) for 4 weeks (*n* = 10 per group). Representative images of liver and lung, and corresponding H&E staining, were shown. (K and L) Dot plots showing the liver/body weight ratio (K) and metastatic foci (L) from the indicated treatment groups in (J). (M) AKT/NRAS-induced HCC mouse models were administered CD and NPD for 4 weeks (*n* = 10 per group). Representative images of liver and lung, and corresponding H&E staining, were shown. (N and O) Dot plots showing the liver/body weight ratio (N) and metastatic foci (O) from the indicated treatment groups in (M). Data are presented as mean ± SD (A to C, E, F, H, I, K, L, N, and O). *P* values were calculated using one-way ANOVA (A to C), Student’s *t* test (E, F, I, K, and N), and Mann–Whitney *U* test (H, L, and O). CD, control diet; Ctrl, control; DAPI, 4′,6-diamidino-2-phenylindole; GFP, green fluorescent protein; NPD, proline-free diet; SD, standard deviation.

To study the pivotal role of l-proline in HCC metastasis, HCC cells were treated with different concentrations of l-proline. Analysis of the l-proline content revealed that the level of l-proline in the medium gradually decreased and remained stable after 24 h post-supplementation; the level of l-proline in cells gradually increased and remained stable after 24 h (Fig. S3A and B). Thus, we further explored the effect of l-proline on HCC by using the indicated concentrations of proline at 24-h supplementation. Indeed, treatment of in vitro cultured HCC cells (MHCC-97H and HCC-LM3) with l-proline, rather than the uncommon natural form (d-proline), promoted their migration and invasion across the tested concentrations, as evidenced by Transwell and wound-healing assays (Fig. 2A and Fig. S3C). The invasion was further confirmed by F-actin staining and three-dimensional (3D) tumor spheroid invasion assays (Fig. 2B and C and Fig. S3D and E).

Next, we investigated whether elevated proline levels are critical for HCC metastasis. In the orthotopic HCC mouse model, the AKT/NRAS-induced HCC mouse model, and the H22 spleen injection liver metastasis mouse model, intraperitoneal administration of 500 mg/kg l-proline increased intratumoral proline levels, whereas treatment with NPD reduced these levels (Fig. S3F). The orthotopic HCC mouse model was intraperitoneally injected with 500 mg/kg l-proline after implantation. l-Proline administration promoted tumor lung metastasis (Fig. 2D to F). Furthermore, the orthotopic HCC mouse model was fed with an NPD or a CD. Consistently, prolonged dietary deprivation of proline was associated with reduced lung metastasis (Fig. 2G to I), again indicating that proline primarily affects HCC metastasis. These findings were corroborated in other HCC models. In the AKT/NRAS-induced HCC mouse model, l-proline administration again promoted lung metastasis (Fig. 2J to L), while proline deprivation suppressed it (Fig. 2M to O). Similarly, in the H22 spleen injection liver metastasis model, l-proline supplementation increased hepatic tumor burden, whereas dietary proline restriction decreased it (Fig. S3G and H). Furthermore, to substantiate that l-proline is a key driver of HCC metastasis, orthotopic HCC mouse models were fed with NPD and administered l-proline supplementation at defined time points. Importantly, l-proline supplementation produced an effect opposite to the metastasis-inhibitory effect of l-proline deprivation, and this effect changed over time. Specifically, early-stage supplementation (especially week 2) dramatically restored metastatic progression, whereas late-stage supplementation (week 4) exerted only minimal effects (Fig. S3I and J). Collectively, these results demonstrated that l-proline promotes HCC metastasis.

### l-Proline specifically binds EPRS1 at the Glu^1123^ residue within the PARS1 domain

**Fig. 3.**
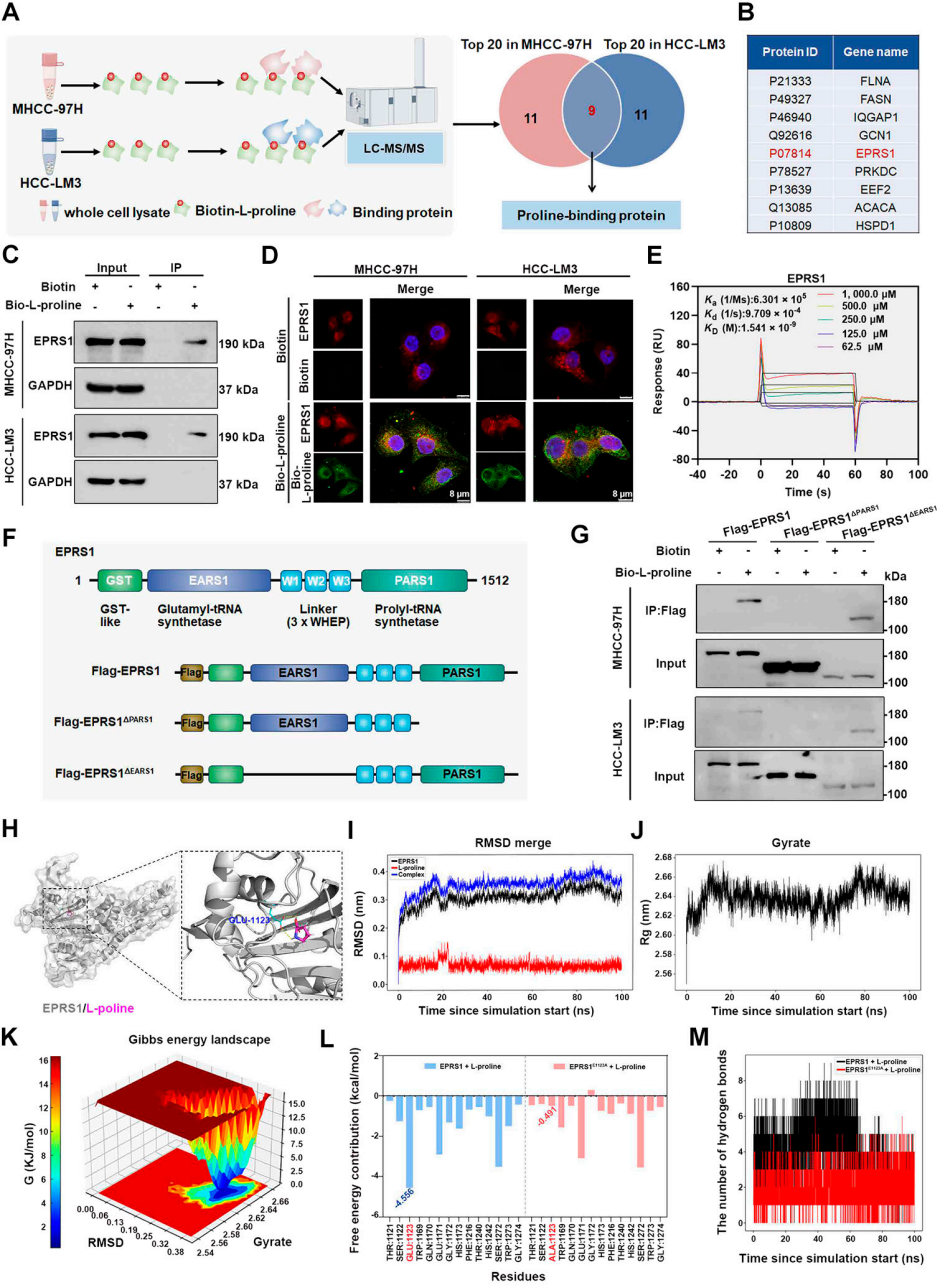
l-Proline specifically binds EPRS1 at the Glu^1123^ residue within the PARS1 domain. (A and B) Schematic of the proteomics workflow for identifying potential l-proline-binding proteins through l-proline pull-down in MHCC-97H and HCC-LM3 cells. (A) Comparison of the top 20 potential l-proline-binding proteins in MHCC-97H and HCC-LM3 cells revealed 9 overlapping candidate binding proteins. (B) Table showing the 9 potential l-proline-binding proteins. EPRS1 is the protein of interest in this study (highlighted in red). (C) Pull-down assay showing the interaction between EPRS1 and bio-l-proline in MHCC-97H and HCC-LM3 cells. Representative blot was shown from 3 biologically independent experiments, and GAPDH was used as a loading control. (D) Immunofluorescence staining showing the colocalization of EPRS1 (red) and bio-l-proline (green) in MHCC-97H and HCC-LM3 cells. Nuclei were counterstained with DAPI (blue) (*n* = 3 per group). (E) SPR showing the interaction between EPRS1 and l-proline at various concentrations (62.5, 125.0, 250.0, 500.0, and 1,000.0 μM). Binding affinity and kinetics were assessed by monitoring the change in RU over time. (F) Schematic diagram of the domains within EPRS1 (upper panel), as well as the full-length (Flag-EPRS1) and deletion mutants (Flag-EPRS1^ΔPARS1^ or Flag-EPRS1^ΔEARS1^; lower panel). (G) Pull-down assay showing the interaction between Flag-tagged full-length or deletion mutants of EPRS1 and bio-l-proline in MHCC-97H and HCC-LM3 cells. (H) Schematic showing the binding site of l-proline within the EPRS1 protein and a predicted key residue, Glu^1123^. (I to K) Molecular dynamics simulations of the EPRS1 and l-proline were run for 100 ns using the AMBER99SB-ILDN force field. (I) RMSD analysis of the molecular dynamics trajectories over time for the EPRS1–l-proline complex (blue line), unbound EPRS1 protein (black line), and free l-proline (red line) to evaluate conformational stability. (J) Rg analysis was performed to evaluate the overall compactness and structural stability of the EPRS1–l-proline complex. (K) Free energy landscape of the EPRS1–l-proline complex was constructed using RMSD and Gyrate as reaction coordinates, which illustrates the stable binding conformation of the complex. (L) Per-residue free energy contribution analysis was performed using the MM/GBSA method, showing the contribution of individual amino acid residues (selected within 4 Å of l-proline) in wild-type EPRS1 (blue) and the EPRS1^E1123A^ mutant (pink) to l-proline binding. (M) Number of hydrogen bonds between l-proline and EPRS1, and EPRS1^E1123A^. ACACA, acetyl-Coa carboxylase α; Bio, biotin-labeled; DAPI, 4′,6-diamidino-2-phenylindole; EARS1, glutamyl-tRNA synthetase 1; EEF2, eukaryotic elongation factor 2; EPRS1, glutamyl-prolyl-tRNA synthetase 1; EPRS1^E1123A^, EPRS1 with the E1123A mutation; FASN, fatty acid synthase; Flag-EPRS1, Flag-tagged full-length EPRS1; Flag-EPRS1^ΔEARS1^, Flag-tagged EPRS1 mutant lacking the EARS1 domain; Flag-EPRS1^ΔPARS1^, Flag-tagged EPRS1 mutant lacking the PARS1 domain; FLNA, filamin A; GCN1, general control non-derepressible protein 1; HSPD1, heat shock protein family D member 1; IP, immunoprecipitation; IQGAP1, IQ motif containing GTPase activating protein 1; PARS1, prolyl-tRNA synthetase 1; PRKDC, protein kinase, DNA-activated, catalytic subunit; Rg, radius of gyration; RMSD, root mean square deviation; RU, response units; SPR, surface plasmon resonance.

Interestingly, recent studies have indicated that amino acids may interact with corresponding binding proteins to regulate tumor development [9]. We hypothesized that this mechanism may also be involved in l-proline-mediated HCC metastasis. To identify potential l-proline-binding proteins, we performed pull-down experiments in MHCC-97H and HCC-LM3 cell lysates. LC-MS/MS analysis identified the top 20 potential l-proline-binding proteins in each cell line, of which 9 were shared between MHCC-97H and HCC-LM3 cell lysates (Fig. 3A and B). In addition, pull-down experiments revealed that 5 of the 9 candidates could bind to l-proline in MHCC-97H and HCC-LM3 cells (Fig. S4A). Next, we knocked down the 5 candidate binding proteins in MHCC-97H cells and subjected the cells to Transwell assay. Knocking down *EPRS1* markedly suppressed MHCC-97H cell migration and invasion (Fig. S4B to D). Thus, we focused on EPRS1 for further study.

To confirm the interaction between l-proline and EPRS1, we first measured baseline proline and EPRS1 levels in PHHs, hepatoblastoma cell line (HepG2), and HCC cell lines (MHCC-97H and HCC-LM3). Compared to PHHs, both proline and EPRS1 expression levels were increased in HepG2, MHCC-97H, and HCC-LM3 cells, especially in MHCC-97H and HCC-LM3 cells (Fig. S4E and F). Then, the interaction of l-proline with EPRS1 in PHHs, hepatoblastoma, and HCC cells was determined by pull-down. The results demonstrated that l-proline–EPRS1 binding occurred in PHHs, hepatoblastoma, and HCC cells (Fig. 3C and Fig. S4F). Consistently, the colocalization of l-proline with EPRS1 in HCC cells was first confirmed by immunofluorescence (Fig. 3D). More importantly, the SPR experiment indicated that l-proline binds to EPRS1 (Fig. S4G), and concentration-gradient assays further revealed a dose-dependent binding response over 62.5 to 1,000.0 μM (Fig. 3E). These data, together with the pull-down and colocalization, support the binding of l-proline to EPRS1. EPRS1 contains a glutamyl-tRNA synthetase 1 (EARS1) domain and a PARS1 domain (Fig. 3F). To identify the l-proline-binding region of EPRS1, we transfected cells with plasmids to overexpress recombinant fragments of EPRS1, including full-length EPRS1 (Flag-EPRS1), a proline-binding domain deletion mutant (Flag-EPRS1^ΔPARS1^), and a glutamate-binding domain deletion mutant (Flag-EPRS1^ΔEARS1^). Only full-length EPRS1 or EPRS1^ΔEARS1^, the fragment containing the PARS1 domain, bound to l-proline (Fig. 3G). This finding was further supported by SPR analysis of the PARS1 domain using a concentration gradient of l-proline (62.5 to 1,000.0 μM), which showed a concentration-dependent interaction (Fig. S4H). We subsequently conducted molecular dynamics simulations to elucidate the binding site of l-proline in EPRS1 (Fig. 3H). The stability of the RMSD, Rg values, and Gibbs energy landscape confirmed the effective binding of l-proline to EPRS1 (Fig. 3I to K). Per-residue energy contribution further identified Glu^1123^ (E1123) as a major contributor to the binding free energy (Fig. 3L). To determine whether this residue is critical for binding stability, an alanine substitution (EPRS1^E1123A^) was generated, and 100-ns molecular dynamics simulations were performed. Compared with the wide-type EPRS1, EPRS1^E1123A^ showed a reduced energy contribution at residue 1123 (Fig. 3L) and a reduced number of hydrogen bonds during the simulation (Fig. 3M), indicating compromised stability of the EPRS1–l-proline interaction. Taken together, these results suggested that l-proline specifically binds EPRS1 at the Glu^1123^ residue within the PARS1 domain.

Given that EPRS1 is a bifunctional tRNA synthetase that catalyzes the attachment of glutamate and proline to cognate transfer RNA for subsequent protein synthesis [35], the pull-down experiment confirmed that, similar to l-proline, glutamate–EPRS1 binding also occurred in HCC cells (Fig. S4I). To investigate whether EPRS1-mediated HCC metastasis is dependent on l-proline or glutamate binding, we transfected HCC cells with a Flag-EPRS1, Flag-EPRS1^ΔPARS1^, and Flag-EPRS1^ΔEARS1^ plasmid to achieve EPRS1 overexpression. The result showed that overexpression of EPRS1 via transfection with the Flag-EPRS1 plasmid enhanced HCC cell migration and invasion, dependent on the PARS1 domain, not EARS1 (Fig. S4J to L). Furthermore, we transfected cells with the plasmids expressing proline-binding site mutant EPRS1^E1123A^ and the glutamate-binding site mutant EPRS1^E205G^ [36] to achieve EPRS1 overexpression. The result showed that the pro-metastatic effect of EPRS1 specifically relied on the l-proline-binding residue Glu^1123^ within the PARS1 domain (Fig. S4M to O). Overall, our study demonstrated that the l-proline–EPRS1 axis specifically promotes metastasis-related phenotypes in HCC.

### l-Proline-bound EPRS1 controls the efficient translation of proteins containing PP motifs

**Fig. 4.**
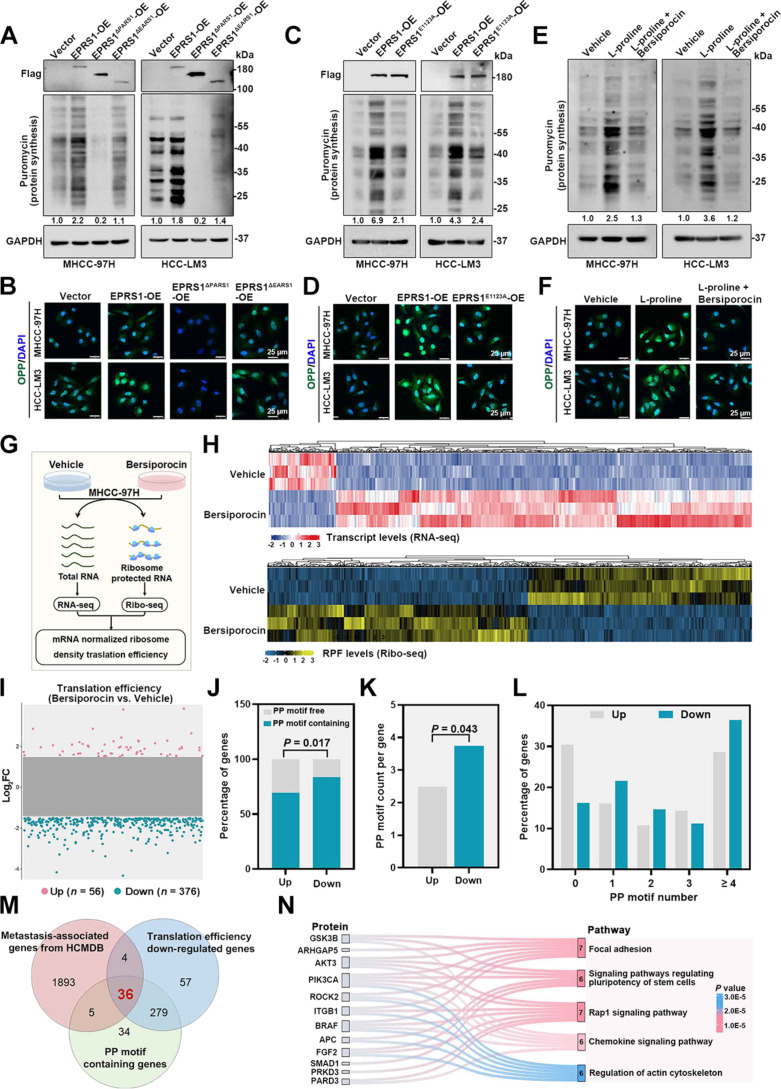
l-Proline-bound EPRS1 controls mRNA translation. (A) Western blotting showing the protein levels of Flag and puromycin incorporation in MHCC-97H and HCC-LM3 cells under different treatment conditions (vector, transfected with vector plasmid; EPRS1-OE, transfected with Flag-EPRS1 plasmid to achieve EPRS1 overexpression; EPRS1^ΔPARS1^-OE, transfected with Flag-EPRS1^ΔPARS1^ plasmid to achieve EPRS1^ΔPARS1^ overexpression; EPRS1^ΔEARS1^-OE, transfected with Flag-EPRS1^ΔEARS1^ plasmid to achieve EPRS1^ΔEARS1^ overexpression). (B) Representative fluorescence images showing the in vitro OPP incorporation (green) and DAPI (blue, nuclei) from the indicated treatment conditions in (A). (C) Western blotting showing the protein levels of Flag and puromycin incorporation in MHCC-97H and HCC-LM3 cells under different treatment conditions (vector, transfected with vector plasmid; EPRS1-OE, transfected with Flag-EPRS1 plasmid to achieve EPRS1 overexpression; EPRS1^E1123A^ -OE, transfected with Flag-EPRS1^E1123A^ plasmid to achieve EPRS1^E1123A^ overexpression). (D) Representative fluorescence images showing the in vitro OPP incorporation (green) and DAPI (blue, nuclei) from the indicated treatment conditions in (C). (E) Western blotting showing the protein levels of puromycin incorporation in MHCC-97H and HCC-LM3 cells following treatment with l-proline (1 mM, 24 h) or the combination of l-proline and bersiporocin (10 μM, 24 h). (F) Representative fluorescence images showing the in vitro OPP incorporation (green) and DAPI (blue, nuclei) from the indicated treatment conditions in (E). (G) Schematic showing the RNA-seq and Ribo-seq workflow to assess TE. MHCC-97H cells treated with or without bersiporocin (10 μM, 24 h) were processed for RNA-seq and Ribo-seq (*n =* 3 per group). (H) Heatmaps showing transcript levels (RNA-seq) and RPF abundance (Ribo-seq) for the indicated treatment conditions (G). (I) Scatter plots showing changes in TE between MHCC-97H cells treated with or without bersiporocin. Each dot represents a gene. Genes with |Log_2_FC| > 1.5 and *P* < 0.05 are colored, with up-regulated genes shown in pink (*n* = 56) and down-regulated genes in blue (*n* = 376). (J) Stacked bar graphs showing the proportions of PP motif-containing and PP motif-free genes in the TE up-regulated and down-regulated groups. (K) Bar graph showing the average number of PP motifs per gene in the TE up-regulated and down-regulated groups. (L) Bar graphs showing the distribution of TE up-regulated and down-regulated genes based on the number of PP motifs (0, 1, 2, 3, ≥4). (M) Venn diagram showing the overlapping genes among 3 gene sets: metastasis-associated genes from the HCMDB database [29] (*n* = 1,938), TE down-regulated genes (I; *n* = 376), and PP motif-containing genes identified from genes with altered TE (*n* = 354). (N) KEGG signaling pathway analysis was performed on the set of overlapping genes, with the top 5 enriched pathways and their corresponding enriched genes shown. The numbers in the box represent the number of genes enriched in each pathway. Representative blot was shown from 3 biologically independent experiments, and GAPDH was used as a loading control (A, C, and E). Data are presented as means (K). *P* values were calculated using chi-square test for (J) and Mann–Whitney *U* test for (K). AKT3, AKT serine/threonine kinase 3; APC, adenomatous polyposis coli; ARHGAP5, Rho GTPase activating protein 5; BRAF, B-Raf proto-oncogene, serine/threonine kinase; DAPI, 4′,6-diamidino-2-phenylindole; EARS1, glutamyl-tRNA synthetase 1; EPRS1, glutamyl-prolyl-tRNA synthetase 1; FC, fold change; FGF2, fibroblast growth factor 2; Flag-EPRS1, Flag-tagged full-length EPRS1; Flag-EPRS1^ΔEARS1^, Flag-tagged EPRS1 mutant lacking the EARS1 domain; Flag-EPRS1^E1123A^, Flag-tagged EPRS1 mutant with the E1123A mutation; Flag-EPRS1^ΔPARS1^, Flag-tagged EPRS1 mutant lacking the PARS1 domain; GAPDH, glyceraldehyde-3-phosphate dehydrogenase; GSK3B, glycogen synthase kinase 3β; HCMDB, human cancer metastasis database; ITGB1, integrin subunit β1; KEGG, Kyoto Encyclopedia of Genes and Genomes; OE, overexpression; OPP, o-propargyl-puromycin; PARD3, par-3 family cell polarity regulator; PARS1, prolyl-tRNA synthetase 1; PIK3CA, phosphatidylinositol-4,5-bisphosphate 3-kinase catalytic subunit α; PP, proline–proline; PRKD3, protein kinase D3; Ribo-seq, ribosome profiling; RNA-seq, RNA sequencing; ROCK2, Rho associated coiled-coil containing protein kinase 2; RPF, ribosome-protected mRNA fragment; SMAD1, SMAD family member 1; TE, translation efficiency.

To investigate the downstream mechanisms by which the l-proline-bound EPRS1 regulates HCC metastasis, we performed correlation analysis. The results revealed that translation was positively correlated with the proline biosynthetic process but negatively correlated with the proline catabolic process (Fig. S5A). Next, we assessed the impact of l-proline-bound EPRS1 on global protein translation in HCC cells. Protein synthesis was inhibited by *EPRS1* knockout (Fig. S5B and C). Conversely, *EPRS1* overexpression enhanced protein synthesis, which was dependent on the l-proline-binding site Glu^1123^ within the PARS1 domain (Fig. 4A to D). Notably, the increase in protein synthesis induced by l-proline in PHHs, hepatoblastoma, and HCC cells was abolished by bersiporocin (Fig. 4E and F and Fig. S5D), an EPRS1 inhibitor that specifically blocks l-proline binding to EPRS1 (Fig. S5E). Since prolyl-tRNA synthetase (PRS) activity in mammalian cells is carried out by both the cytosolic enzyme EPRS1 and the mitochondrial enzyme PARS2, we further evaluated whether bersiporocin also affects PARS2. PARS2 plays an important role in mitochondrial protein synthesis [37]. To evaluate its function, we measured ATP levels, the activities of mitochondrial respiratory chain complex I and IV, and mitochondrial protein abundance in HCC cells treated with bersiporocin. All these parameters remained unchanged, indicating that the PARS2 function was not impaired (Fig. S5F to I). Based on the potential of l-proline-bound EPRS1 in protein synthesis, we next performed RNA-seq and Ribo-seq of MHCC-97H cells with or without bersiporocin exposure (Fig. 4G). Bersiporocin exposure altered the gene transcription and ribosome-protected mRNA fragment (RPF) abundance (Fig. 4H and Fig. S6A and B). By comparing TE in MHCC-97H cells with or without bersiporocin treatment, we found that 56 mRNAs had increased TE and 376 mRNAs had decreased TE (Fig. 4I).

Given this discrepancy, we next sought to confirm whether l-proline and EPRS1 regulate a specific subset of mRNA targets rather than the global transcriptome. Previous studies have found that during the synthesis of proteins containing consecutive proline, such as Pro–Pro (PP), Pro–Pro–Gly (PPG), Pro–Pro–Pro (PPP), or longer proline strings, ribosomes often stall during translation elongation [38,39]. Therefore, we hypothesize that the translation of genes bearing more PP motifs is more sensitive to bersiporocin, resulting in a decreased TE. Consistent with this hypothesis, compared to the TE up-regulated group, the TE down-regulated group contained a higher proportion of PP motif-containing genes (Fig. 4J) and genes in this group exhibited a greater number of PP motifs per gene (Fig. 4K), with a higher proportion containing ≥4 PP motifs (Fig. 4L). Taken together, these results demonstrated that bersiporocin treatment preferentially reduced the TE of genes containing PP motifs. Given that bersiporocin specifically inhibits proline binding to EPRS1, these findings suggest that genes containing PP motifs are likely preferential targets of the l-proline and EPRS1 axis.

To screen specific downstream effectors of l-proline and EPRS1 as potential target genes against HCC metastasis, we intersected 3 datasets and identified 36 metastasis-associated genes containing PP motifs whose translation was down-regulated after bersiporocin treatment (Fig. 4M). Moreover, KEGG analysis revealed that 12 PP motif-containing genes were enriched in the top 5 metastasis-associated pathways (Fig. 4N and Fig. S6C). We next knocked down these 12 candidate genes in MHCC-97H cells and performed Transwell assays. We found that knockdown of *PIK3CA*, *AKT3*, *ITGB1*, *FGF2*, and *PARD3* markedly suppressed MHCC-97H cell migration and invasion (Fig. S6D and E).

**Fig. 5.**
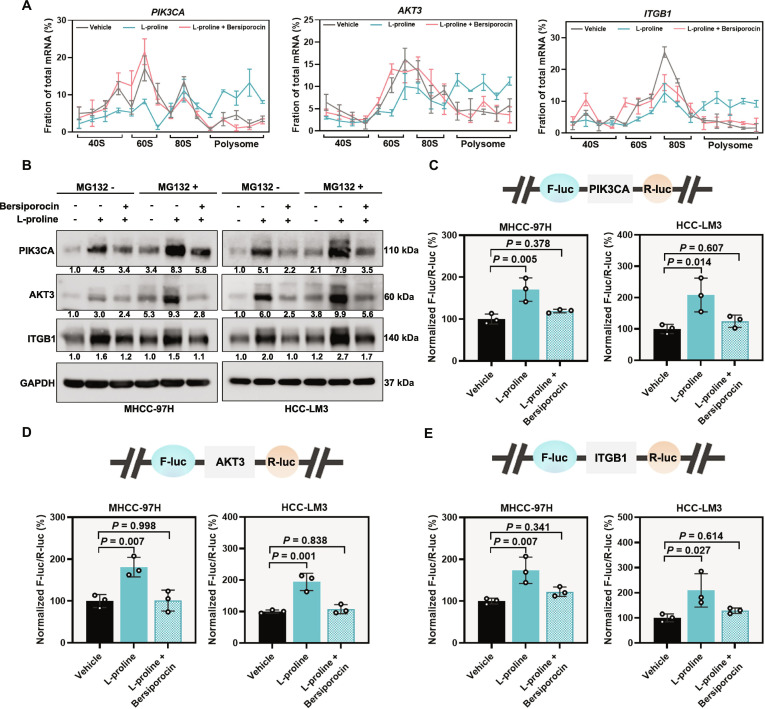
l-Proline-bound EPRS1 controls the translation of PIK3CA, AKT3, and ITGB1. (A) RT-qPCR analysis of the relative mRNA distributions of *PIK3CA*, *AKT3*, and *ITGB1* in ribosome fractions of MHCC-97H cells following treatment with l-proline (1 mM, 24 h) or the combination of l-proline and bersiporocin (10 μM, 24 h; *n =* 3 per group). (B) Western blotting showing the protein levels of PIK3CA, AKT3, and ITGB1 in HCC cell lines treated with l-proline (1 mM, 24 h) or the combination of l-proline and bersiporocin (10 μM, 24 h), followed by treatment with or without 10 μM MG132 for 12 h. Representative blot was shown from 3 biologically independent experiments, and GAPDH was used as a loading control. (C to E) Bar graphs showing the TE of *PIK3CA*, *AKT3*, and *ITGB1* in HCC cell line treated with l-proline (1 mM, 24 h) or the combination of l-proline and bersiporocin (10 μM, 24 h), followed by transfection with pmirGLO-PIK3CA, pmirGLO-AKT3, or pmirGLO-ITGB1 reporters for 24 h. TE was defined as reporter protein production (F-luc/R-luc) divided by mRNA abundance (*n =* 3 per group). (C to E) Data are presented as mean ± SD, and *P* values were calculated using one-way ANOVA. AKT3, AKT serine/threonine kinase 3; F-luc, firefly luciferase; GAPDH, glyceraldehyde-3-phosphate dehydrogenase; ITGB1, integrin subunit β1; PIK3CA, phosphatidylinositol-4,5-bisphosphate 3-kinase catalytic subunit α; R-luc, renilla luciferase; SD, standard deviation.

To validate the TE of these 5 candidate genes, we performed a polysome profiling experiment. The results showed that l-proline only promoted the TE of *PIK3CA, AKT3,* and *ITGB1* mRNAs, whereas bersiporocin inhibited their translation (Fig. 5A and Fig. S7A). Moreover, we examined the effect of l-proline on protein translation in the HCC mouse model. Expectedly, l-proline administration enhanced translation of *PIK3CA, AKT3*, and *ITGB1* in vivo, whereas l-proline deprivation inhibited it (Fig. S7B to I). Furthermore, to determine whether bersiporocin reduces PIK3CA, AKT3, and ITGB1 protein levels by promoting their degradation, we treated HCC cells with the proteasome inhibitor MG132. MG132 treatment did not restore the protein expression of these genes in bersiporocin-treated cells (Fig. 5B), suggesting that their down-regulation is not mediated by enhanced proteasomal degradation. Thus, we hypothesized that the decreased expression levels of these 3 proteins might stem from variations in TE. To verify this, we generated the pmirGLO-PIK3CA, pmirGLO-AKT3, and pmirGLO-ITGB1 luciferase reporter plasmids. The dual luciferase assay revealed a marked increase in the TE of *PIK3CA*, *AKT3*, and *ITGB1* in l-proline-treated cells, which was reversed by bersiporocin (Fig. 5C to E). Collectively, these data indicated that EPRS1, when bound to l-proline, regulates the efficient translation of *PIK3CA*, *AKT3*, and *ITGB1*.

### PIK3CA, AKT3, and ITGB1 are required contributors for EPRS1-induced HCC metastasis

**Fig. 6.**
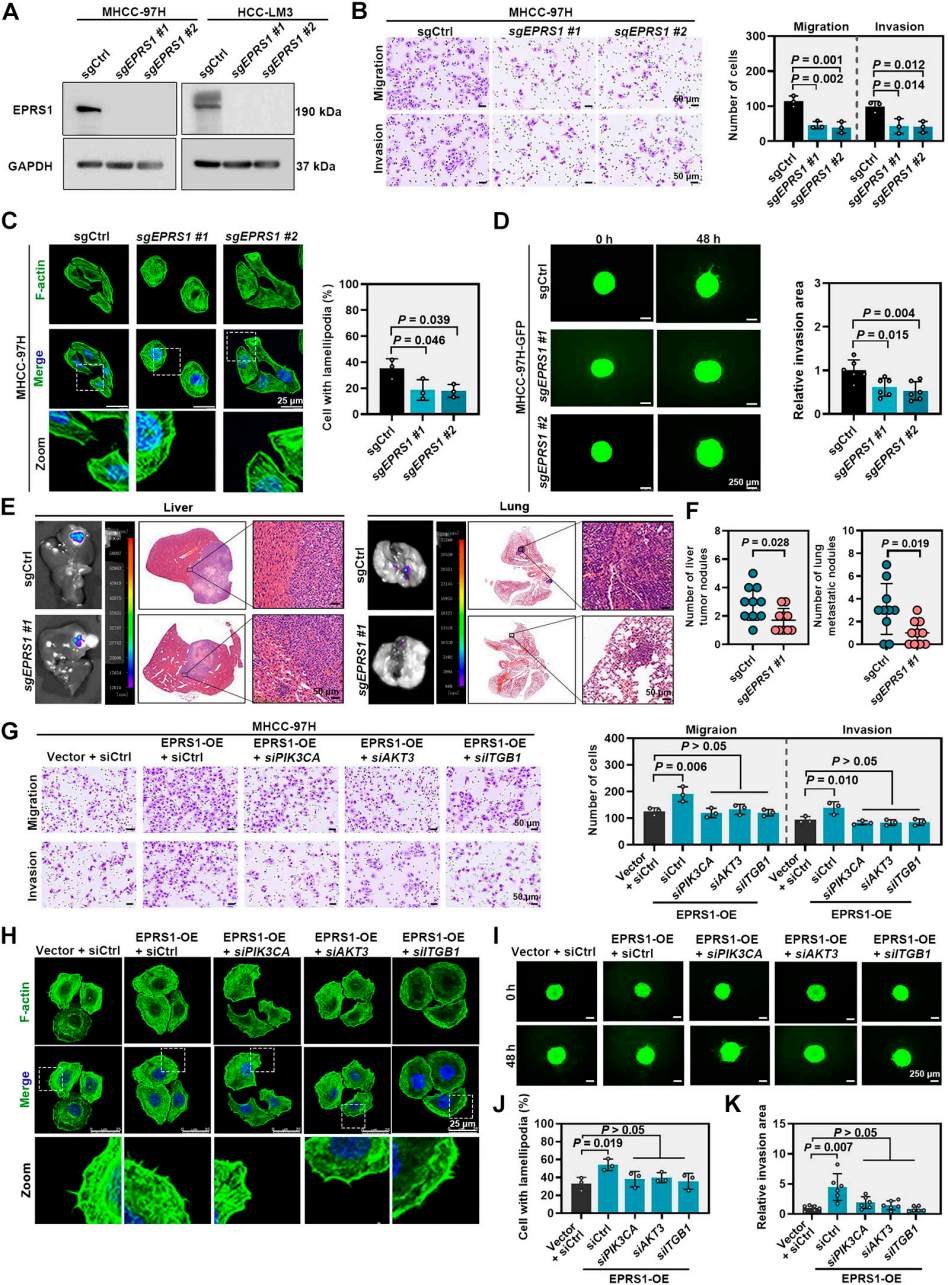
PIK3CA, AKT3, and ITGB1 are responsible for EPRS1-induced HCC metastasis. (A) Western blotting showing the protein levels of EPRS1 in *EPRS1*-knockout (*sgEPRS1* #1 and *sgEPRS1* #2) or control (sgCtrl) MHCC-97H and HCC-LM3 cells. Representative blot was shown from 3 biologically independent experiments, and GAPDH was used as a loading control. (B) Left: Transwell assays showing the migratory and invasive abilities in sgCtrl, *sgEPRS1* #1, and *sgEPRS1* #2 MHCC-97H cells. The quantification data are shown on the right (*n* = 3 per group). (C) Left: Representative immunofluorescence images of F-actin organization in sgCtrl, *sgEPRS1* #1, and *sgEPRS1* #2 MHCC-97H cells. Cells were stained with phalloidin (green, F-actin) and DAPI (blue, nuclei). The quantification data are shown on the right (*n* = 3 per group). (D) Left: Representative images showing the in vitro 3D invasion in sgCtrl, *sgEPRS1* #1, and *sgEPRS1* #2 MHCC-97H-GFP cells. The quantification data are shown on the right (*n* = 6 per group). Images were captured at 0 and 48 h after the spheroids were embedded into the invasion matrix. (E) *sgCtrl or sgEPRS1 #1* MHCC-97H cells were orthotopically injected into the BALB/c nude mice (*n =* 10 per group). Representative fluorescence images of liver and lung, and corresponding H&E staining, were shown. (F) Dot plots showing the tumor nodules of the liver (left) and the lung (right) from the indicated treatment groups in (E). (G) Left: Transwell assays showing the migratory and invasive abilities of MHCC-97H cells under different treatment conditions (vector + siCtrl, transfected with vector plasmid and siCtrl; EPRS1-OE + siCtrl, transfected with Flag-EPRS1 plasmid and siCtrl; EPRS1-OE + *siPIK3CA*, transfected with Flag-EPRS1 plasmid and *siPIK3CA*; EPRS1-OE + *siAKT3*, transfected with Flag-EPRS1 plasmid and *siAKT3*; EPRS1-OE + *siITGB1*, transfected with Flag-EPRS1 plasmid and *siITGB1*). The quantification data are shown on the right (*n* = 3 per group). (H) Representative immunofluorescence images of F-actin organization from the indicated treatment groups in (G). MHCC-97H cells were stained with phalloidin (green, F-actin) and DAPI (blue, nuclei; *n* = 3). (I) Representative images showing the in vitro 3D invasion of MHCC-97H-GFP cells from the indicated treatment groups in (G) (*n* = 6 per group). Images were captured at 0 and 48 h after the spheroids were embedded into the invasion matrix. (J and K) Quantification data of F-actin organization (J) and in vitro 3D invasion (K) in (H) and (I). Data are presented as mean ± SD, and *P* values were calculated using one-way ANOVA (B to D, G, and J), Mann–Whitney *U* test (F, left panel), Student’s *t* test (F, right panel), and Kruskal–Wallis (K). AKT3, AKT serine/threonine kinase 3; DAPI, 4′,6-diamidino-2-phenylindole; EPRS1, glutamyl-prolyl-tRNA synthetase 1; Flag-EPRS1, Flag-tagged full-length EPRS1; GAPDH, glyceraldehyde-3-phosphate dehydrogenase; ITGB1, integrin subunit β1; OE, overexpression; PIK3CA, phosphatidylinositol-4,5-bisphosphate 3-kinase catalytic subunit α; SD, standard deviation.

EPRS1 expression was elevated in EHM tissues compared with that in NM tissues (Fig. S8A to D). To investigate whether EPRS1 is important for HCC metastasis, we knocked out *EPRS1* in HCC cells (Fig. 6A). Transwell assays revealed that *EPRS1* depletion attenuated HCC cell migration and invasion (Fig. 6B and Fig. S8E), which was further verified by F-actin staining and 3D tumor spheroid invasion assays (Fig. 6C and D and Fig. S8F and G). Moreover, *EPRS1*-knockout MHCC-97H cells were orthotopically transplanted into the livers of BALB/c nude mice to assess the effect of EPRS1 on HCC metastasis in vivo. We found fewer metastases in the knockout group than in the control group (Fig. 6E and F). The above data indicate that EPRS1 promotes HCC metastasis*.*

To confirm that PIK3CA, AKT3, and ITGB1 are necessary for EPRS1-induced HCC metastasis, we first examined the expression levels of *PIK3CA*, *AKT3*, and *ITGB1* in tumor tissues from the orthotopic HCC mouse model established with MHCC-97H cells expressing *sgEPRS1*. Compared with the control group, the mRNA levels of *PIK3CA*, *AKT3*, and *ITGB1* remained stable, while their protein levels were markedly reduced (Fig. S8H and I). Then, puromycin incorporation assays confirmed that *EPRS1* deletion suppressed protein translation (Fig. S8J). Next, we silenced these genes in *EPRS1-*overexpressing cells. *EPRS1* overexpression markedly promoted the migration and invasion of HCC cells. However, individual silencing of *PIK3CA*, *AKT3*, or *ITGB1* effectively inhibited this promoting effect (Fig. 6G to K and Fig. S9A to C), indicating that each of these genes is required for EPRS1-induced pro-metastatic phenotypes. Moreover, l-proline-induced HCC cell metastasis phenotypes were abolished by individual silencing of *PIK3CA*, *AKT3*, or *ITGB1* (Fig. S9D to I). In summary, we found that each of these genes, *PIK3CA*, *AKT3*, and *ITGB1*, is responsible for EPRS1-induced metastasis-related phenotypes in HCC.

### l-Proline-bound EPRS1 is a therapeutic target for HCC metastasis

**Fig. 7.**
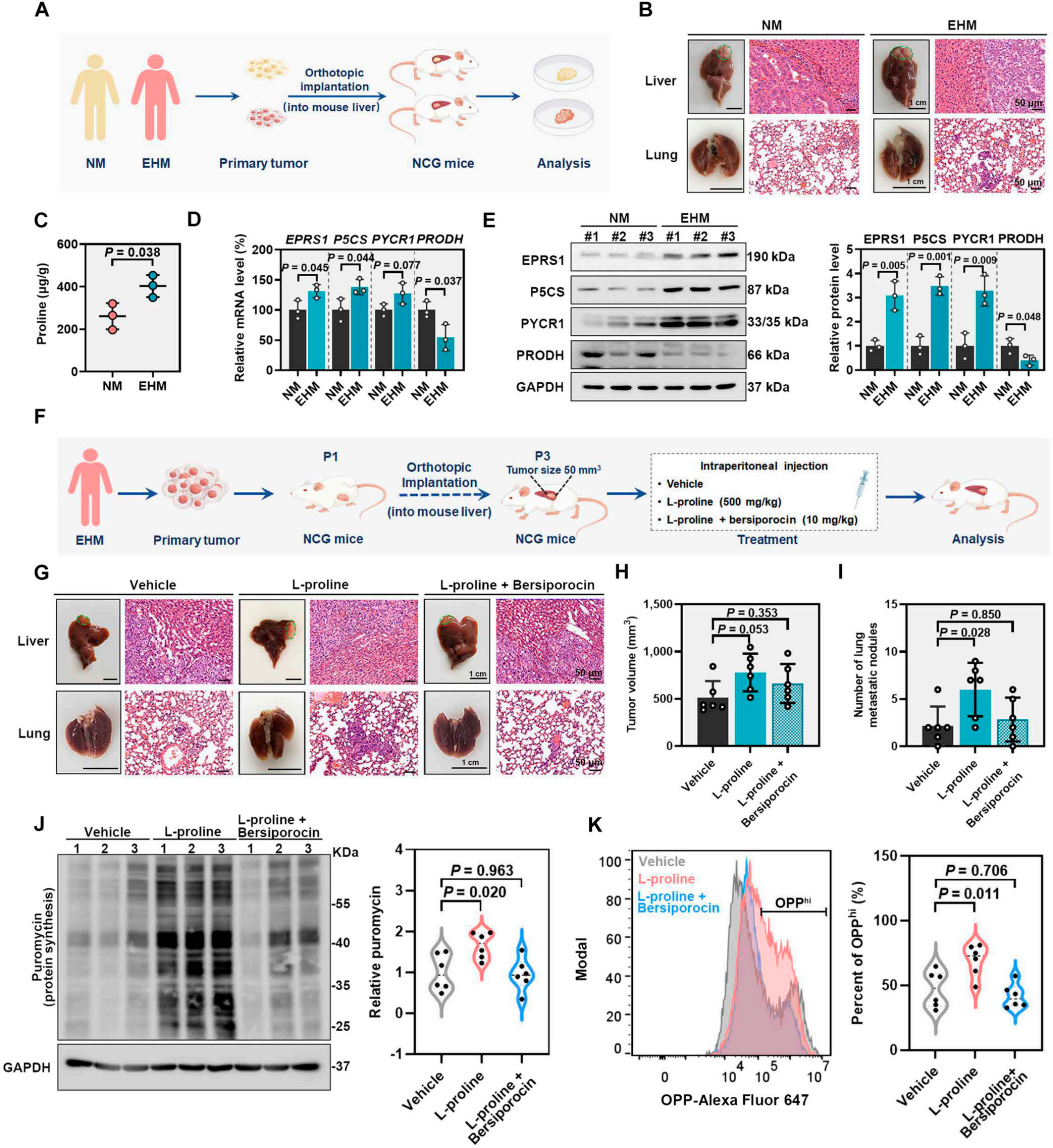
l-Proline-bound EPRS1 is a therapeutic target for HCC metastasis. (A) Schematic of HCC PDOX model construction. Primary liver tumors from NM and EHM patients were orthotopically implanted into the mouse liver to establish HCC PDOX models for downstream analysis (*n* = 3 per group). (B) Representative images of liver and lung, and corresponding H&E staining from NM- and EHM-derived PDOX models. (C) Dot plots showing the proline levels quantified by a colorimetric assay in liver tumor tissues from NM- and EHM-derived PDOX models. (D) RT-qPCR analysis of the mRNA levels of *EPRS1*, *P5CS*, *PYCR1*, and *PRODH* in liver tumor tissues from PDOX models (*n* = 3 per group). (E) Western blotting showing the protein levels of EPRS1, P5CS, PYCR1, and PRODH in liver tumor tissues from PDOX models. The quantification data are shown on the right. GAPDH was used as a loading control. (F) Schematic of the PDOX model construction and therapeutic intervention. Following orthotopic implantation of the liver tumor, NCG mice were treated intraperitoneally every 2 d with vehicle (DMSO in PBS), l-proline (500 mg/kg), or the combination of l-proline and bersiporocin (10 mg/kg) for 4 weeks (*n* = 6 per group). P1 and P3 indicate passage 1 and passage 3 of the xenograft models, respectively. (G) Representative images and corresponding H&E staining of liver and lung from the EHM-derived PDOX model in (F), which received the indicated treatment groups. (H and I) Bar charts showing the liver tumor volume (H) and number of metastatic nodules (I) from the EHM-derived PDOX model in (F), which received the indicated treatment groups. (J) Western blotting showing the protein levels of puromycin incorporation in tumor cells from the EHM-derived PDOX model in (F), which received the indicated treatment groups. GAPDH was used as a loading control. The quantification data are shown on the right (*n* = 6). (K) Representative histogram plot showing the OPP flow cytometric analysis of tumor cells extracted from the EHM-derived PDOX model in (F), which received the indicated treatment groups. The quantification data are shown on the right (*n* = 6). GAPDH was used as a loading control (E and J). Data are presented as mean ± SD, and *P* values were calculated using Student’s *t* test (C to E), Kruskal–Wallis (H), and one-way ANOVA (I to K). EHM, extrahepatic metastasis; EPRS1, glutamyl-prolyl-tRNA synthetase 1; GAPDH, glyceraldehyde-3-phosphate dehydrogenase; NCG, NOD/ShiLtJGpt-Prkdc^em26Cd52^Il2rg^em26Cd22^/Gpt; NM, no metastasis; OPP, o-propargyl-puromycin; PDOX, patient-derived orthotopic xenograft; P1, passage 1; P3, passage 3; P5CS, pyrroline-5-carboxylate synthetase; PBS, phosphate-buffered saline; PRODH, proline dehydrogenase; PYCR1, pyrroline-5-carboxylate reductase 1; SD, standard deviation.

To comprehensively evaluate the impact of l-proline on HCC metastasis in vivo, we first established a PDOX mouse model by using HCC tissues from EHM or NM (Fig. 7A). Mice bearing EHM-derived tumors (the EHM group) exhibited larger tumor volumes compared to those bearing NM-derived tumors (the NM group; Fig. 7B and Fig. S10A). Notably, the level of proline in the EHM group was markedly higher than that in the NM group (Fig. 7C). Consistently, increased EPRS1, P5CS, and PYCR1 expression and decreased PRODH expression were observed in tumors from the EHM group compared with those from the NM group (Fig. 7D and E), suggesting an association between altered proline metabolism and HCC metastasis.

Bersiporocin demonstrated a general safety profile in a first-in-human phase I clinical trial [40]. In animals, it exhibited promising pharmacokinetics (*C*_max_ = 841.93 ng/ml, half-life = 2.18 h) and showed no marked toxicity in major organs or in liver function markers (Fig. S10B to D). To evaluate the role of bersiporocin in inhibiting HCC metastasis, we constructed EHM-derived PDOX tumor models in NCG mice (Fig. 7F). Compared with vehicle treatment, l-proline treatment enhanced lung metastasis and was accompanied by increased intertumoral proline levels (Fig. 7G to I and Fig. S10E). Notably, administration of the EPRS1 inhibitor bersiporocin attenuated l-proline-induced promotion of HCC metastasis (Fig. 7G to I). To assess the effect of l-proline on protein synthesis in vivo, tumor cells were isolated from PDOX mice. Western blotting and flow cytometry analyses confirmed that l-proline treatment enhanced protein synthesis, and this effect was abolished by bersiporocin (Fig. 7J and K and Fig. S10F). Consistently, the expression of the downstream molecules PIK3CA, AKT3, and ITGB1 was increased by l-proline and suppressed by bersiporocin (Fig. S10G). Based on these findings, we explored combination therapies in the EHM-derived PDOX model. Notably, the administration of bersiporocin in combination with either the PI3K inhibitor alpelisib or the AKT inhibitor MK-2206 reduced the tumor volume and the number of lung metastatic nodules (Fig. S10H to J). Taken together, we proposed that l-proline-bound EPRS1 exerts a pivotal role in HCC metastasis.

## Discussion

Currently, marked efforts are devoted to understanding and addressing the complexities of metastatic liver cancer, which constitutes a major barrier to effective treatment development. Although an increasing number of studies have indicated that reprogramming of amino acid metabolism critically regulates tumor metastasis [41], the specific amino acids that determine HCC metastasis remain to be identified. Here, by integrating proteomics and targeted metabolomics based on clinical cohorts and orthotopic HCC mouse models, we demonstrated that proline metabolism reprogramming plays a crucial role in HCC metastasis. Prior studies have primarily examined proline metabolism as a static nutrient source for HCC proliferation [16] and tumor initiation [42], which is derived from external uptake or glutamate/ornithine conversion [43]. In our study, we revealed that l-proline fluctuation actively drives HCC metastasis rather than merely representing a passive byproduct through dynamic tracking of amino acid pools. Moreover, we uncovered a mechanism whereby l-proline directly binds EPRS1 to promote tumor dissemination, beyond its established roles in collagen formation [34] and redox regulation [33]. Importantly, we identified l-proline-binding proteins as critical metastatic mediators, suggesting a potential strategy for therapeutic targeting that extends beyond simply inhibiting proline synthesis or uptake. Collectively, these findings established that l-proline was a pivotal molecule that orchestrates HCC metastasis via a specific EPRS1 interaction mechanism, offering a promising direction for therapeutic intervention.

Amino acid restriction therapy has shown certain therapeutic effects in diseases, especially in tumors [44]. A recent study revealed that a valine-restricted diet in combination with PARP inhibitor treatment enhances antitumor effects in colorectal cancer [45]. Similarly, restricting the intake of serine and glycine has been shown to markedly inhibit the proliferation of intestinal cancer and lymphoma [46]. In the context of liver cancer, restriction of tryptophan intake can prevent MYC-dependent tumorigenesis [47], indicating that amino acid restriction may be a potential therapeutic approach for HCC management. Notably, we revealed that l-proline restriction is an effective strategy for inhibiting HCC metastasis. Consistently, existing studies have emphasized that cancer cells obtain proline, which fuels HCC proliferation and initiates tumor development, both from the external environment and via the metabolic conversion of glutamate or ornithine [43]. Preliminary evidence of the potential mechanism underlying proline-mediated tumorigenesis has been provided by earlier observations. Proline is a key component of the ECM, and the abundance of proline and collagen directly influences the plasticity and heterogeneity of cancer cells [34]. In addition to its role in collagen synthesis, proline is also closely associated with redox homeostasis [33] and epithelial–mesenchymal transition [48], a critical process by which tumor cells gain migratory and invasive capabilities. Moreover, studies have indicated that proline metabolism can enhance the stemness of breast cancer [49], prostate cancer [50], and pancreatic ductal adenocarcinoma [51], thereby impacting their metastatic potential. Here, our findings established that l-proline drives HCC metastasis via a binding-dependent mechanism involving EPRS1, rather than through its classical roles in collagen synthesis or redox balance.

EPRS1, which functions as a bifunctional tRNA synthetase, plays an essential role in catalyzing the ligation of glutamate and proline to their corresponding transfer RNAs, facilitating subsequent protein synthesis [35]. An increasing number of studies have indicated that EPRS1 expression is abnormally elevated in a multitude of cancers, such as breast cancer [18], gastric cancer [52], and neuroblastoma [53], and is closely associated with a poor prognosis. In HCC, EPRS1 is frequently up-regulated, and this phenotype is correlated with shortened patient survival [19]. Interestingly, our research revealed that EPRS1 can promote HCC metastasis by binding to l-proline. Metabolite–protein interactions have been recently recognized to be more prevalent than previously appreciated [54], and in certain instances, these interactions can have marked functional consequences. For example, cholesterol promotes oral squamous cell carcinoma cell migration by binding with caveolin 1, which further regulates its cellular localization [55]. Additionally, the tricarboxylic acid cycle intermediate succinate can bind to hypoxia-inducible factor 1 subunit α (HIF-1α), leading to increased stabilization of HIF-1α [56]. Our research found that l-proline can bind to EPRS1 and control the translation of proteins containing PP motifs, among which PIK3CA, AKT3, and ITGB1 are closely related to HCC metastasis. Depletion of these 3 genes also impaired EPRS1-mediated HCC metastasis, indicating that EPRS1 promotes HCC progression by regulating the translation of specific proteins. These findings are consistent with other findings that EPRS1 can regulate the proline-rich proteins laminin subunit γ1 (LAMC1) and cyclin B1 (CCNB1) to promote the malignant progression of HCC [19]. Thus, interfering with the l-proline–EPRS1 interaction to inhibit the translation of specific proteins may be one of the therapeutic approaches for HCC.

Bersiporocin is an EPRS1 inhibitor that selectively targets PRS. Its innovative mechanism involves asymmetric binding to the PARS1 domain within EPRS1, effectively inhibiting PRS activity without completely impairing the enzyme’s function [40]. Using the EPRS1 inhibitor bersiporocin, we discovered that the EPRS1–proline axis acts as a translational control pathway to regulate metastasis-associated mRNA translation and HCC metastasis. Bersiporocin has primarily been evaluated in research with a focus on translational modulation in fibrotic organs, such as the lung and kidney. It was reported that bersiporocin showed good antifibrotic effects in idiopathic pulmonary fibrosis, and this unique inhibitor had been approved for clinical treatment [40]. Moreover, the therapeutic potential of bersiporocin has also been demonstrated in tubulointerstitial nephritis, in which it prevents idiopathic pulmonary fibrosis and alleviates immunofibrotic aggravation [57]. However, little data are available in the context of oncology. Our finding was that bersiporocin interferes with the binding of l-proline to EPRS1, which led to suppression of the translation of metastasis-associated genes, thereby inhibiting HCC metastasis. Overall, disrupting the synthesis of proteins that promote cancer development via the use of EPRS1 inhibitors may be a promising approach for HCC management.

Meanwhile, the present study has several limitations. First, although our study focused on proline metabolism, we observed that several other amino acids were also up-regulated during HCC metastasis. While we had established the role of proline metabolism in metastasis, the individual contributions and potential synergies among these altered amino acids remain to be systematically investigated. Second, while our multistep validation supports the role of EPRS1 in l-proline binding, the screen for l-proline-binding proteins was not exhaustive; thus, undiscovered interactors may exist and warrant further investigation. Finally, while bersiporocin appears specific to HCC metastasis, its optimal long-term dosing regimen has not been determined. Although phase I trials in healthy subjects and our preclinical studies provided preliminary pharmacokinetic and safety references, clinical evidence in HCC patients was still lacking to define an appropriate treatment strategy. Further studies are warranted to address this issue.

## Conclusions

Our findings indicated that the level of proline, which was closely associated with HCC metastasis, was increased in HCC patient tumor samples, HCC mouse models, and HCC cell lines. Modulation of proline level influences the metastasis of HCC. Notably, we found that l-proline controls the translation of metastasis-related genes by binding to EPRS1. In addition, we showed the inhibitory effects of bersiporocin on this function. In summary, our studies suggested a better understanding of l-proline-mediated HCC metastasis, and these findings had implications in the development of treatment strategies to combat tumor metastasis.

## Ethical Approval

All experiments involving animals were conducted in accordance with the Chinese National Animal Welfare Ethics Review Guidelines (GB/T 35892-2018). Animal research followed the guiding principles of “Animal Research: In vivo Experiment Report” (ARRIVE) and was approved by the Animal Ethics Committee of Chongqing Medical University (IACUC-CQMU-2024-0202). All procedures involving human sample collection and application were approved by the Ethics Committee of Chongqing Medical University (approval number: 2024061) and Tianjin Medical University Cancer Institute and Hospital (approval number: bc20254421). Informed consent was obtained from all the participants. The study conforms to the principles outlined in the Declaration of Helsinki.

## Data Availability

The RNA-seq and Ribo-seq analysis data supporting the results of this study have been deposited in the NCBI Sequence Read Archive (SRA) database (accession IDs: PRJNA1211022 and PRJNA1211181). The mass spectrometry proteomic data in this study have been deposited in the ProteomeXchange Consortium database (dataset identifier, PXD059613). The data that support the findings of this study are available from the corresponding authors on reasonable request.
