# Supplementary material for: Dysregulated Proline Metabolism Exacerbates Hepatocellular Carcinoma Metastasis via EPRS1-Mediated mRNA Translation
Source: Cancer Commun (Lond). 2026 May 14;46:0028. doi: 10.34133/cancomm.0028 (PMC13172588; doi:10.34133/cancomm.0028)
Supplement: Supplementary 1 — Figs. S1 to S10 Tables S1 to S7 [file cancomm.0028.f1.pdf]

Supplementary Materials of

Dysregulated proline metabolism exacerbates hepatocellular carcinoma metastasis via EPRS1-mediated mRNA translation

Hui Zhang[1,2,†], Leirong Gu[2,†], Xiameng Su[2,†], Wanjin Chen[2], Ming Tan[3], Haibo Yu[2], Hongzhong Zhou[4], Tingting Gao[2], Zhiling Wang[2], Xinyan Chen[2], Weixian Chen[1], Juan Chen[1,2,*], Shengtao Cheng[1,2,*]

Affiliations

[1]Department of Laboratory Medicine, Key Laboratory of Clinical Laboratory Diagnostics (Ministry of Education), The Second Affiliated Hospital, Chongqing Medical University, Chongqing, P. R. China.

[2]Department of Infectious Diseases, Key Laboratory of Molecular Biology for Infectious Diseases (Ministry of Education), Institute for Viral Hepatitis, The Second Affiliated Hospital, Chongqing Medical University, Chongqing, P. R. China.

[3]Department of Laboratory Medicine, Sichuan Provincial People's Hospital, University of Electronic Science and Technology of China, Chengdu, Sichuan, P. R. China.

[4]Department of Laboratory Medicine, Shenzhen Institute of Translational Medicine, The First Affiliated Hospital of Shenzhen University, Shenzhen Second People’s Hospital, Health Science Center, Shenzhen University, Shenzhen, Guangdong, P. R. China.

[†]Hui Zhang, Leirong Gu, and Xiameng Su contributed equally to this work.

[*]Corresponding authors:

Shengtao Cheng

Department of Laboratory Medicine, Key Laboratory of Clinical Laboratory Diagnostics (Ministry of Education), The Second Affiliated Hospital, Chongqing Medical University, Chongqing 400010, P. R. China. E-mail: shengtao@cqmu.edu.cn.

Juan Chen

Department of Laboratory Medicine, Key Laboratory of Clinical Laboratory Diagnostics (Ministry of Education), The

Second Affiliated Hospital, Chongqing Medical University, Chongqing 400010, P. R. China. E-mail: chenjuan2014@cqmu.edu.cn.

Supplementary Figures

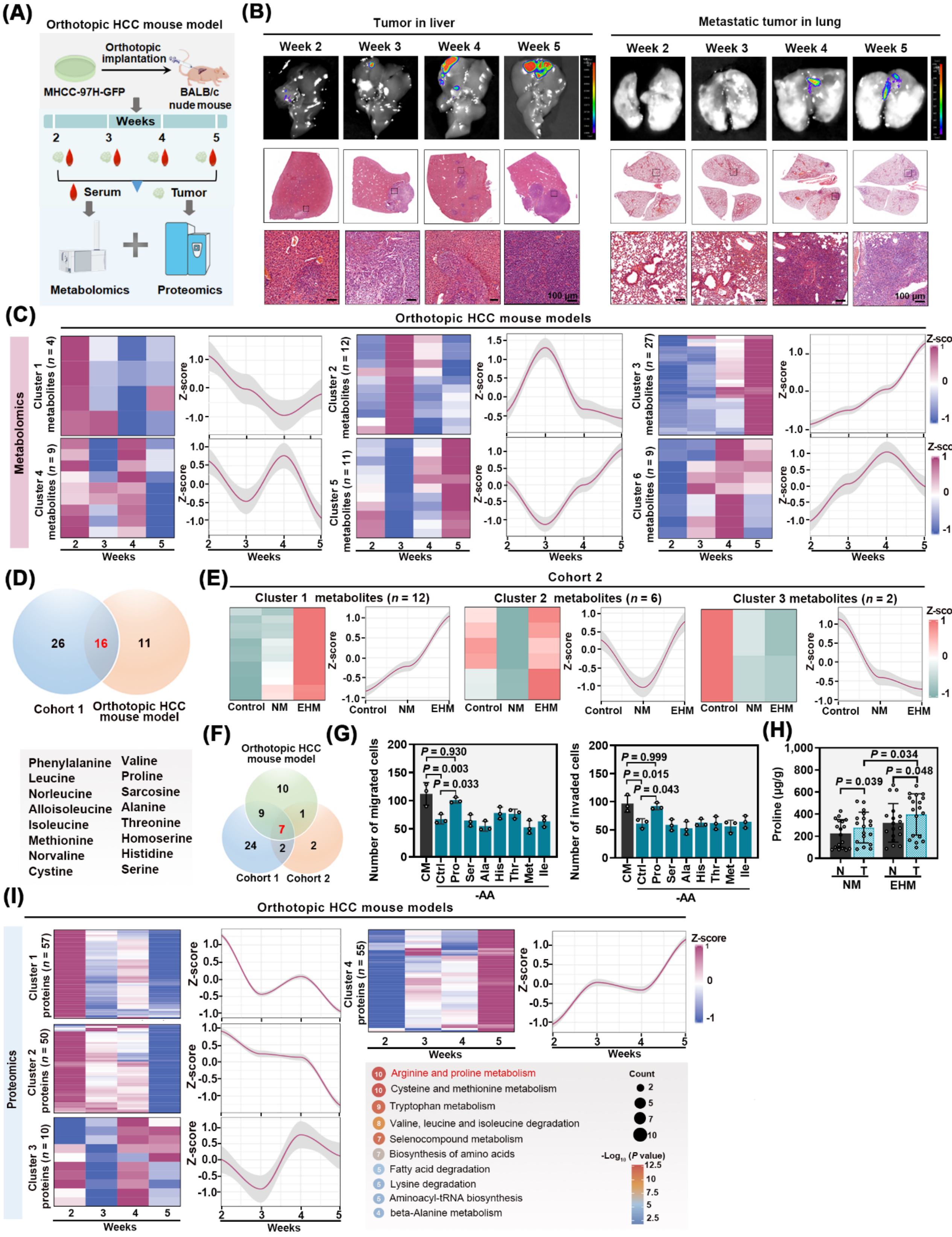

Supplementary Figure S1. Proline metabolic reprogramming in the orthotopic HCC mouse model and in cohort

2.

(A-B) MHCC-97H-GFP cells were orthotopically implanted into BALB/c nude mice to establish orthotopic HCC mouse models (n = 6 per group). Serum and tumor tissues were collected at the indicated weeks (2, 3, 4, and 5) post-implantation for subsequent metabolomics and proteomics analyses (A). Representative fluorescence images of liver and lung (upper panel) and corresponding H&E staining (lower panel) were shown (B). **(C)** Serum amino acid metabolites from the orthotopic HCC mouse models, identified by LC-MS/MS, were clustered based on their z-score normalized expression profiles from week 2 to week 5 post-implantation (n = 5 per group). The number in parentheses indicates the metabolite count for each cluster, and the curves depict the mean z-score trajectory of each cluster across the groups. The gray shadows around the curves represent the 95% confidence intervals of the curves. **(D)** Venn diagram showing that 16 serum amino acid metabolites were identified based on shared consistent up-regulation trends in the orthotopic HCC mouse model (from week 2 to week 5 post-implantation) and cohort 1(from control to NM to EHM). The list of 16 amino acids is provided below. **(E)** Serum amino acid metabolites in cohort 2, identified by LC-MS/MS, were clustered based on their z-score normalized expression profiles across the control (n = 52), NM (n = 116), and EHM (n = 60) groups. The number in parentheses indicates the metabolite count for each cluster, and the curves depict the mean z-score trajectory of each cluster across the groups**.** The gray shadows around the curves represent the 95% confidence intervals of the curves. **(F)** Venn diagram showing that 7 serum amino acid metabolites were identified based on shared consistent up-regulation trends in the orthotopic HCC mouse model (from week 2 to week 5 post-implantation), cohort 1 (from control to NM to EHM group) and cohort 2 (from control to NM to EHM group). **(G)** Transwell assays showing the migratory and invasive abilities of MHCC-97H cells under different treatment conditions (CM, complete medium as positive control; Ctrl-AA, all amino acids depleted DMEM medium as negative control; Pro/Ser/Ala/His/Thr/Met/Ile-AA, all amino acids depleted DMEM medium supplemented with Pro (11.5 mg/L, 24h); Ser (52.5 mg/L, 24h); Ala (8.9 mg/L, 24h); His (42 mg/L, 24h); Thr (95 mg/L, 24h); Met (30 mg/L, 24h); Ile (105 mg/L, 24h) (n = 3 per group). **(H)** Bar chart showing the concentration of proline quantified by a colorimetric assay in paired tumor and adjacent normal tissues from 36 HCC patients in cohort 1 (NM, n = 18; EHM, n = 18). **(I)** Proteins from orthotopic HCC mouse models were identified by LC-MS/MS and filtered for those belonging to the Reactome pathway R-HSA-71291. These proteins were then clustered based on their z-score normalized expression profiles from weeks 2 to 5 post-implantation (n = 6 per group). The number in parentheses indicates the protein count for each cluster, and the curves depict the mean z-score trajectory of each cluster across the groups. The gray shadows around the curves represent the 95% confidence intervals of the curves. KEGG pathway enrichment was performed for cluster 4 proteins (right-lower panel). The number inside the circle represents the number of proteins enriched in each pathway. Data are presented as mean ± SD, and P values were calculated using one-way ANOVA (G) and non-parametric t-tests (H).

Abbreviations: Ala, alanine; CM, complete medium; Ctrl, control; EHM, extrahepatic metastasis; H&E, hematoxylin and eosin; HCC, hepatocellular carcinoma; His, histidine; Ile, isoleucine; Met, methionine; N, adjacent non-tumor liver tissue; NM, no metastasis; Pro, proline; Ser, serine; T, primary HCC tissues; Thr, threonine; -AA, all amino acids depleted DMEM medium.

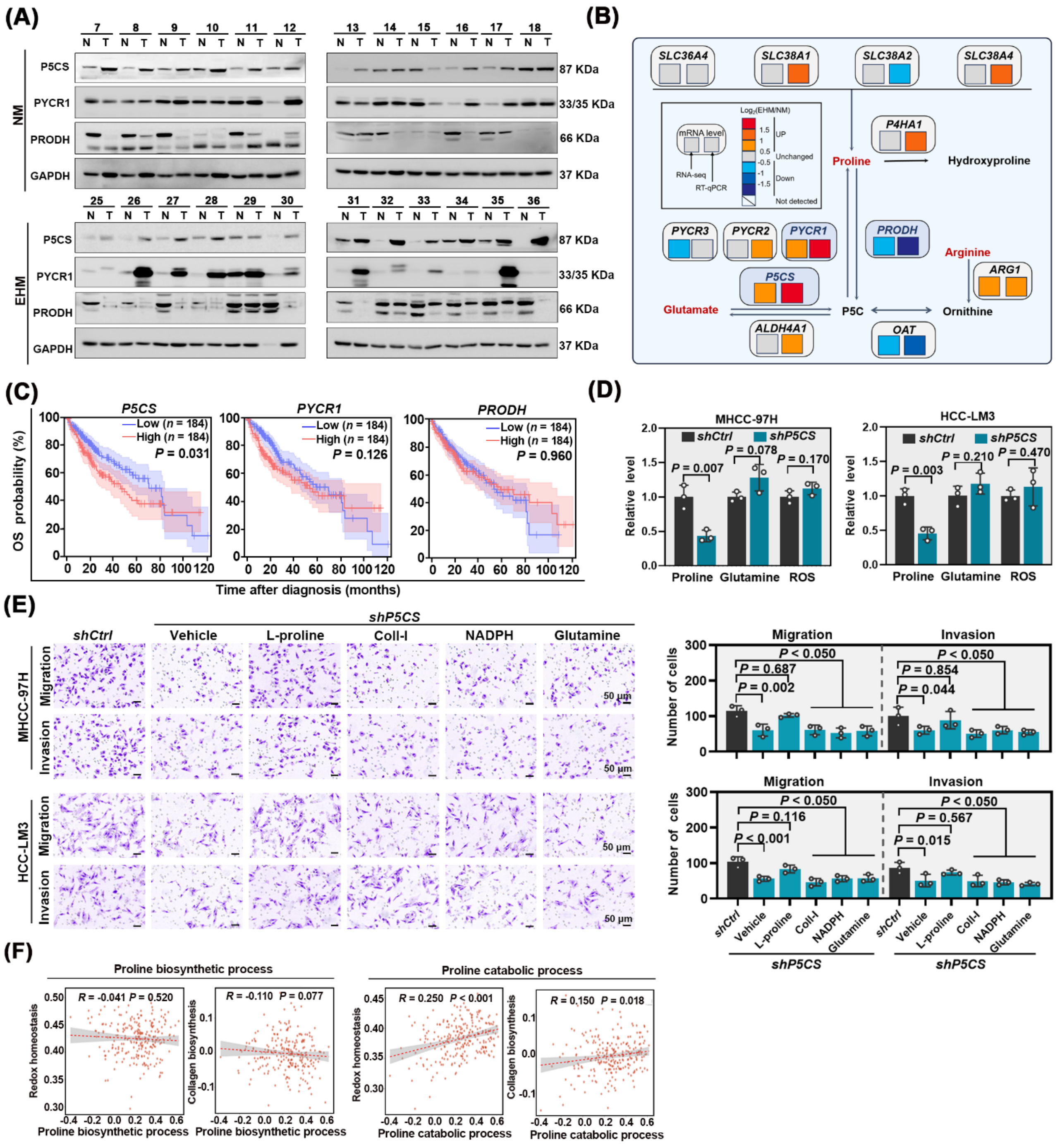

Supplementary Figure S2. Expression levels of proline-metabolizing enzymes and P5CS-mediated metastatic phenotype in HCC cells.

(A) Representative images of Western blotting showing the protein levels of P5CS, PYCR1 and PRODH in paired tumor and adjacent normal tissues from HCC patients in cohort 1 (n = 36). **(B)** Tumor tissues of 17 patients (NM, n = 9; EHM, n = 8) were subjected to RNA sequencing and RT-qPCR. The schematic diagram shows the key genes involved in proline metabolism. For each gene, the colored box below it indicates the $\log_2$(fold change) in mRNA levels in EHM versus NM tumors, based on RNA-seq (left) and RT-qPCR validation (right). The color scale represented the direction and

magnitude of change: red (upregulated), gray (unchanged), blue (downregulated), and white (not detected). The mRNA level of key enzymes in proline metabolism was highlighted with a blue background (*P5CS*, *PYCR1* and *PRODH*). (**C**) OS analysis for the *P5CS*, *PYCR1*, and *PRODH* genes was performed using the TCGA-LIHC dataset via the GEPIA3 database, with the median expression level serving as the cutoff. (**D**) Proline, glutamine and ROS levels were detected in MHCC-97H and HCC-LM3 cells without or with silencing of *P5CS* (n = 3 per group). (**E**) Left, Transwell assays showing the migratory and invasive abilities of MHCC-97H and HCC-LM3 cells under different treatment conditions [*shCtrl*, infected with control lentivirus and supplemented with PBS; *shP5CS* + Vehicle, infected with *shP5CS* lentivirus and supplemented with PBS; *shP5CS* + L-proline, infected with *shP5CS* lentivirus and supplemented with L-proline (1 mmol/L, 24h); *shP5CS* + Coll-I, infected with *shP5CS* lentivirus and supplemented with Coll-I (0.2 μg/mL, 24h); *shP5CS* + NADPH, infected with *shP5CS* lentivirus and supplemented with NADPH (1 mmol/L, 24h); *shP5CS* + glutamine, infected with *shP5CS* lentivirus and supplemented with glutamine (4 mmol/L, 24h)]. The quantification data were shown on the right (n = 3 per group). (**F**) Correlation graphs showing associations between the expression of genes involved in proline metabolism and redox homeostasis or collagen biosynthesis, based on Spearman's rank correlation analysis of the GSE14520 dataset. GAPDH was used as a loading control (A). Data are presented as mean ± SD, and P values were calculated using Student's t-test (D) and one-way ANOVA (E). Abbreviations: ALDH4A1, aldehyde dehydrogenase 4 family member a1; ARG1, arginase 1; Coll-I, collagen type I; Ctrl, control; EHM, extrahepatic metastasis; GEPIA, Gene Expression Profiling Interactive Analysis; HCC, hepatocellular carcinoma; N, adjacent non-tumor liver tissue; NADPH, nicotinamide adenine dinucleotide phosphate; NM, no metastasis; OAT, ornithine aminotransferase; OS, overall survival; P4HA1, prolyl 4-hydroxylase subunit alpha 1; P5C, pyrroline-5-carboxylate; P5CS, pyrroline-5-carboxylate synthetase; PBS, phosphate-buffered saline; RT-qPCR, quantitative fluorescence PCR; PRODH, proline dehydrogenase; PYCR1, pyrroline-5-carboxylate reductase 1; PYCR2, pyrroline-5-carboxylate reductase 2; PYCR3, pyrroline-5-carboxylate reductase 3; RNA-seq, RNA sequencing; ROS, reactive oxygen species; SLC36A4, solute carrier family 36 member 4; SLC38A1, solute carrier family 38 member 1; SLC38A2, solute carrier family 38 member 2; SLC38A4, solute carrier family 38 member 4; T, primary HCC tissues

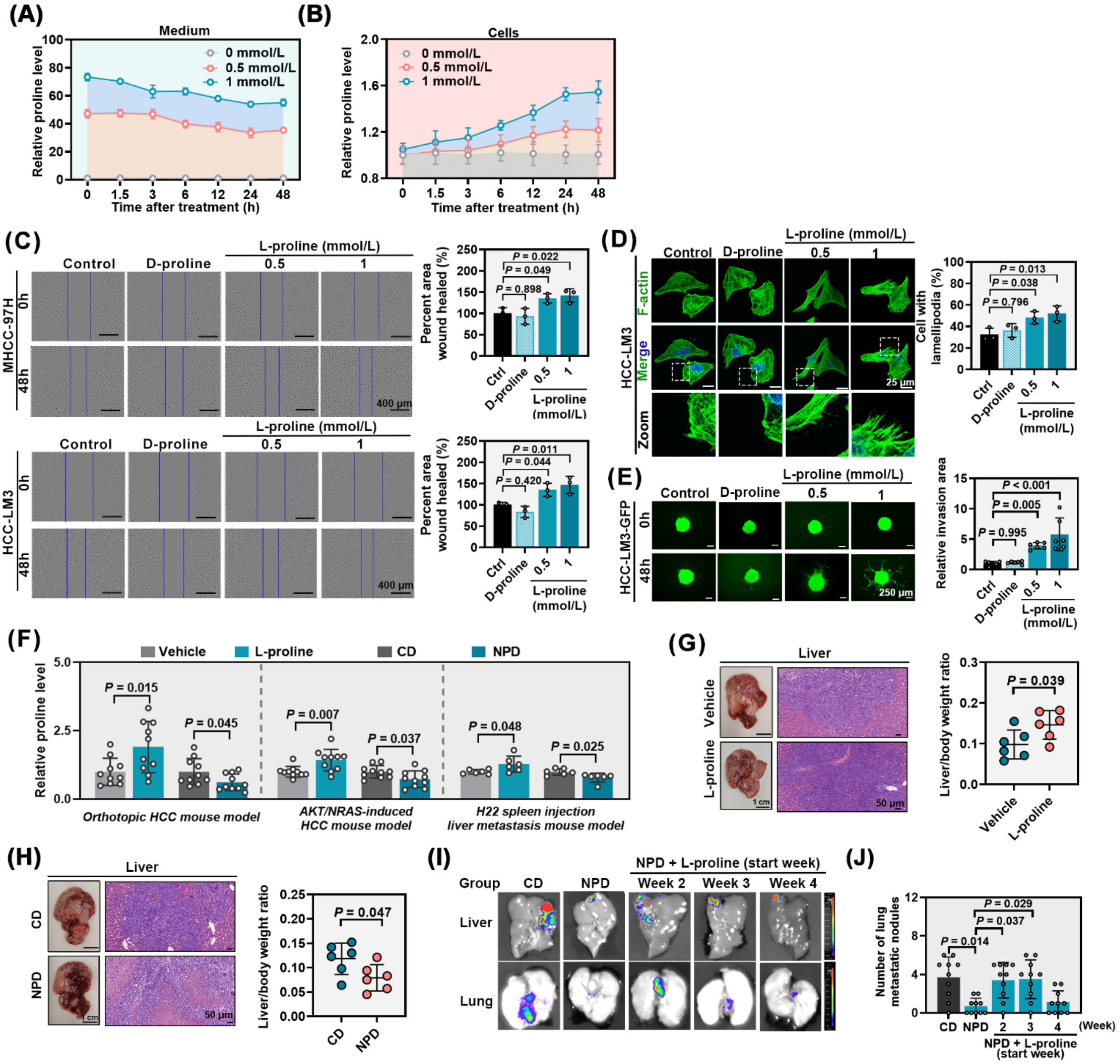

Supplementary Figure S3. L-Proline promotes HCC metastasis.

(A-B) The concentrations of proline in the culture medium (A) and intracellularly (B) were measured in MHCC-97H cells at the indicated time points (0, 1.5, 3, 6, 12, 24, 48 h) after treatment with L-proline at various concentrations (n = 3 per group; 0, 0.5, 1 mmol/L). **(C)** Left, wound healing assays showing the migratory abilities of MHCC-97H and HCC-LM3 cells treated for 24 h with: control (PBS); D-proline (1 mmol/L; negative control); L-proline (0.5 or 1 mmol/L). The quantification data was shown on the right (n = 3 per group). **(D)** Left, representative immunofluorescence images of F-actin organization from the indicated treatment conditions in (C). HCC-LM3 cells were stained with phalloidin (green, F-actin) and DAPI (blue, nuclei). The quantification data are shown on the right (n = 3 per group). **(E)** Left, representative images showing the *in vitro* 3D invasion of HCC-LM3-GFP cells from the indicated treatment

conditions in (C). The quantification data were shown on the right (n = 6 per group). Images were captured at 0 hours and 48 hours after the spheroids were embedded into the invasion matrix. (**F**) Bar chart showing the proline levels in liver tumor tissues from three mouse models: orthotopic HCC mouse model, AKT/NRAS-induced HCC mouse model, and H22 spleen injection liver metastasis mouse model. Mice were treated intraperitoneally every 2 days with either vehicle (PBS) or L-proline (500 mg/kg) and fed with CD or NPD. (**G**) H22 spleen injection liver metastasis models were treated intraperitoneally every 2 days with either vehicle (PBS) or L-proline (500 mg/kg) for 2 weeks (n = 6 per group). Representative images of the liver and corresponding H&E staining were shown (left). Dot plots showing the liver/body weight ratio from the indicated treatment groups (right). (**H**) H22 spleen injection liver metastasis models were administered CD or NPD for 2 weeks (n = 6 per group). Representative images of the liver and corresponding H&E staining were shown (left). Dot plots showing the liver/body weight ratio from the indicated treatment groups (right). (**I-J**) Orthotopic HCC mouse models were treated under five conditions: two control groups were fed either CD or NPD for 5 weeks; three intervention groups were fed the NPD diet and received intraperitoneal injections of L-proline (500 mg/kg, every 2 days) starting at week 2, 3, and 4 post-implantation (NPD feeding was continuous), respectively (n = 10 per group). Representative fluorescence images of liver and lung were shown (I). Bar chart showing the number of lung metastatic nodules from the indicated treatment groups (J). Data are presented as mean ± SD, and P values were calculated using one-way ANOVA (C-E), Student's t-test (F-H) and Kruskal-Wallis (J). Abbreviations: CD, control diet; Ctrl, control; DAPI, 4',6-Diamidino-2-Phenylindole; H&E, hematoxylin and eosin; HCC, hepatocellular carcinoma; NPD, proline-free diet; PBS, phosphate-buffered saline.

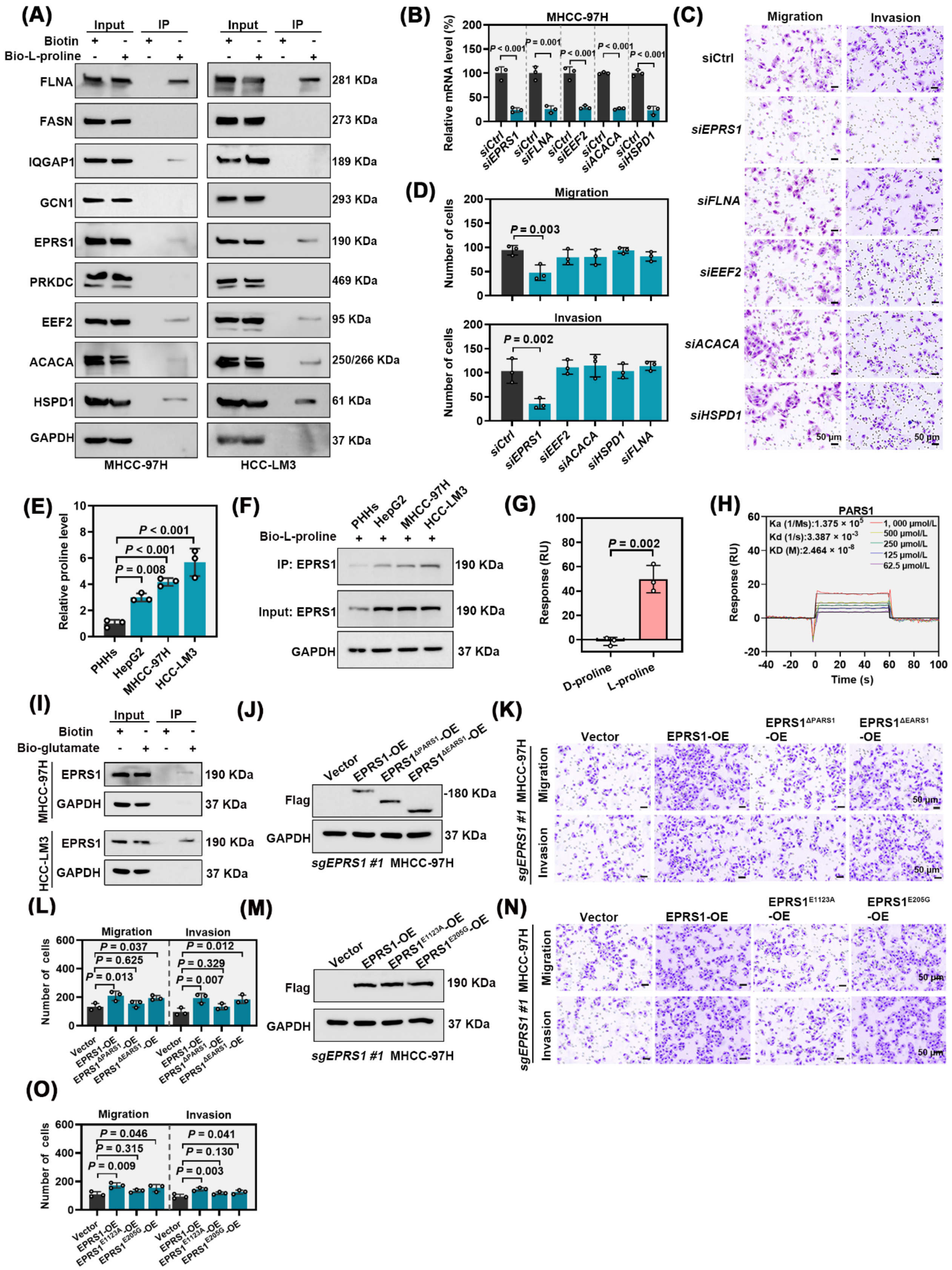

Supplementary Figure S4. EPRS1 is an L-proline-binding protein that promotes metastasis-related phenotypes. **(A)** Pull-down assays showing the interaction of Bio-L-proline with candidate binding proteins (FLNA, FASN, IQGAP1, GCN1, EPRS1, PRKDC, EEF2, ACACA, and HSPD1) in MHCC-97H and HCC-LM3 cells. **(B)** RT-qPCR analysis of

the mRNA levels of *EPRS1, FLNA, EEF2, ACACA* and *HSPD1* in MHCC-97H cells transfected with the corresponding siRNA (siCtrl, *siEPRS1*, *siFLNA*, *siEEF2*, *siACACA* and *siHSPD1*) (n = 3 per group). **(C-D)** Transwell assays showing the migratory and invasive abilities of MHCC-97H that were transfected with the corresponding siRNA (siCtrl, *siEPRS1*, *siFLNA*, *siEEF2*, *siACACA,* and *siHSPD1*) (C). The quantification data was shown (D; n = 3 per group). (**E**) Bar chart showing the relative proline level in PHHs, HepG2, MHCC-97H and HCC-LM3 cells (n = 3 per group). (**F**) Pull-down assay showing the interaction between EPRS1 and Bio-L-proline in PHHs, HepG2, MHCC-97H and HCC-LM3 cells. (**G**) SPR showing the interaction of EPRS1 with D-proline (1 mmol/L) or L-proline (1 mmol/L; n = 3 per group). (**H**) SPR showing the interaction between PARS1 and L-proline at various concentrations (62.5, 125.0, 250.0, 500.0, and 1,000.0 μmol/L). Binding affinity and kinetics were assessed by monitoring the change in resonance units (RU) over time. (**I**) Pull-down assay showing the interaction between EPRS1 and Bio-glutamate in MHCC-97H and HCC-LM3 cells. (**J-L**) *sgEPRS1 #1* MHCC-97H cells under different treatment conditions (vector, transfected with vector plasmid; EPRS1-OE, transfected with Flag-EPRS1 plasmid to achieve EPRS1 overexpression; EPRS1$^{\Delta PARS1}$-OE, transfected with Flag-EPRS1$^{\Delta PARS1}$ plasmid to achieve EPRS1$^{\Delta PARS1}$ overexpression; EPRS1$^{\Delta EARS1}$-OE, transfected with Flag-EPRS1$^{\Delta EARS1}$ plasmid to achieve EPRS1$^{\Delta EARS1}$ overexpression). Western blotting showing the protein levels of Flag (J). Transwell assays showing the migratory and invasive abilities (K). The quantification data was shown (L; n = 3 per group). (**M-O**) *sgEPRS1 #1* MHCC-97H cells under different treatment conditions (vector, transfected with vector plasmid; EPRS1-OE, transfected with Flag-EPRS1 plasmid to achieve EPRS1 overexpression; EPRS1^{E1123A}-OE, transfected with Flag-EPRS1 ^{E1123A} plasmid to achieve EPRS1 ^{E1123A} overexpression; EPRS1^{E205G} -OE, transfected with Flag-EPRS1^{E205G} plasmid to achieve EPRS1^{E205G} overexpression). (M) Western blotting showing the protein levels of Flag (M). Transwell assays showing the migratory and invasive abilities (N). The quantification data was shown (O; n = 3 per group). Representative blot was shown from 3 biologically independent experiments, and GAPDH was used as a loading control (A, F, I-J, M). Data are presented as mean ± SD, and P values were calculated using Student's t-test (B, G) and one-way ANOVA (D-E, L, O). Abbreviations: ACACA, acetyl-Coa carboxylase alpha; Bio, biotin-labeled; Ctrl, control; EARS1, glutamyl-tRNA synthetase 1; EEF2, eukaryotic translation elongation factor 2; EPRS1, glutamyl-prolyl-tRNA synthetase 1; FASN, fatty acid synthase; Flag-EPRS1, Flag-tagged full-length EPRS1; Flag-EPRS1^{E1123A}, Flag-tagged EPRS1 mutant with the E1123A mutation; Flag-EPRS1^{E205G}, Flag-tagged EPRS1 mutant with the E205G mutation; Flag-EPRS1$^{\Delta EARS1}$, Flag-tagged EPRS1 mutant lacking the EARS1 domain; Flag-EPRS1$^{\Delta PARS1}$, Flag-tagged EPRS1 mutant lacking the PARS1 domain; FLNA, filamin A; GAPDH, glyceraldehyde-3-phosphate dehydrogenase; GCN1, general control non-derepressible protein 1; HSPD1, heat shock protein family; IP, immunoprecipitation; IQGAP1, IQ motif containing GTPase activating protein 1; OE, overexpression; PARS1, prolyl-tRNA synthetase 1; PHHs, primary human hepatocytes; PRKDC, DNA-dependent protein kinase catalytic subunit; RT-qPCR, quantitative

fluorescence PCR; RU, response units; SPR, surface plasmon resonance.

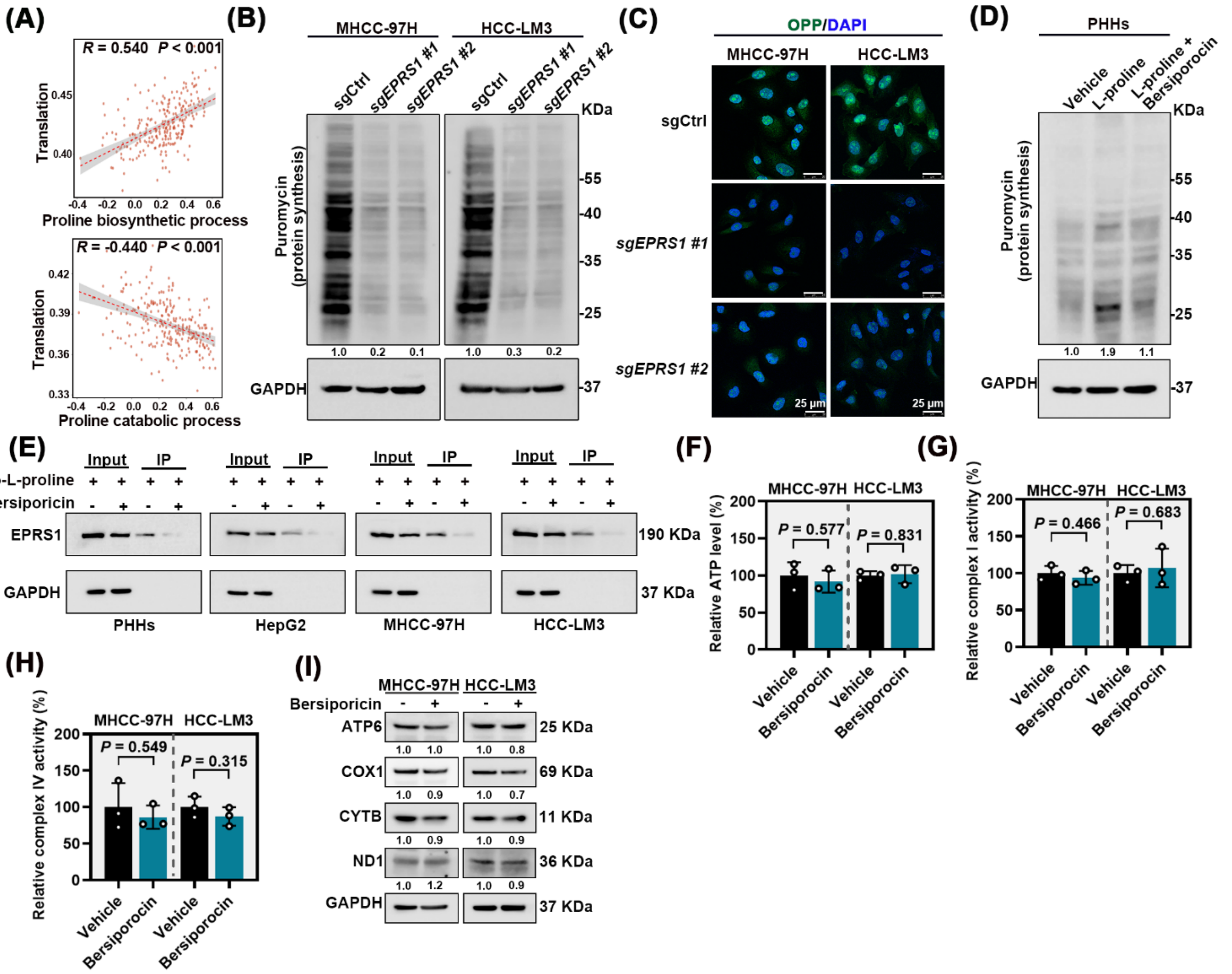

Supplementary Figure S5. The role of EPRS1 in global mRNA translation, and the effects of bersiporocin on EPRS1-proline interaction and mitochondrial function.

(A) Correlation graphs showing associations between the expression of genes involved in proline metabolism and translation based on Spearman's rank correlation analysis of the GSE14520 dataset. **(B)** Western blotting showing the protein levels of puromycin incorporation in control (sgCtrl) and *EPRS1*-knockout (*sgEPRS1* #1 and *sgEPRS1* #2) MHCC-97H and HCC-LM3 cells. **(C)** Representative fluorescence images showing the *in vitro* OPP incorporation (green) and DAPI (blue, nuclei) in control (sgCtrl) or *EPRS1*-knockout (*sgEPRS1* #1 and *sgEPRS1* #2) MHCC-97H and HCC-LM3 cells ($n = 3$). **(D)** Western blotting showing the protein levels of puromycin incorporation in PHHs cells following treatment with L-proline (1 mmol/L, 24h) or the combination of L-proline and bersiporocin (10 μmol/L, 24h). (**E**) Pull-down assay showing the interaction between EPRS1 and Bio-L-proline in PHHs, HepG2, MHCC-97H and HCC-LM3 cells with or without bersiporocin (10 μmol/L, 24h) treatment. (**F-H**) Bar charts showing the level of ATP (F) and the activity of mitochondrial respiratory chain complex I (G) and IV (H) in MHCC-97H and HCC-LM3 cells following treatment with or without bersiporocin (10 μmol/L) ($n = 3$ per group). (**I**) Western blotting showing the protein

level of ATP6, COX1, CYTB and ND1 in MHCC-97H and HCC-LM3 cells following treatment with or without bersiporocin (10 μmol/L). Representative blot was shown from 3 biologically independent experiments, and GAPDH was used as a loading control (B, D, I). Data are presented as mean ± SD, and *P* values were calculated using Student's *t*-test (F-H). Abbreviations: ATP, adenosine triphosphate; ATP6, ATP synthase F0 subunit 6; Bio, biotin-labeled; COX1, cytochrome c oxidase subunit 1; CYTB, cytochrome b; DAPI, 4',6-Diamidino-2-Phenylindole; EPRS1, glutamyl-prolyl-tRNA synthetase 1; GAPDH, glyceraldehyde-3-phosphate dehydrogenase; IP, immunoprecipitation; ND1, NADH dehydrogenase subunit 1; OPP, O-propargyl-puromycin; PHHs, primary human hepatocytes.

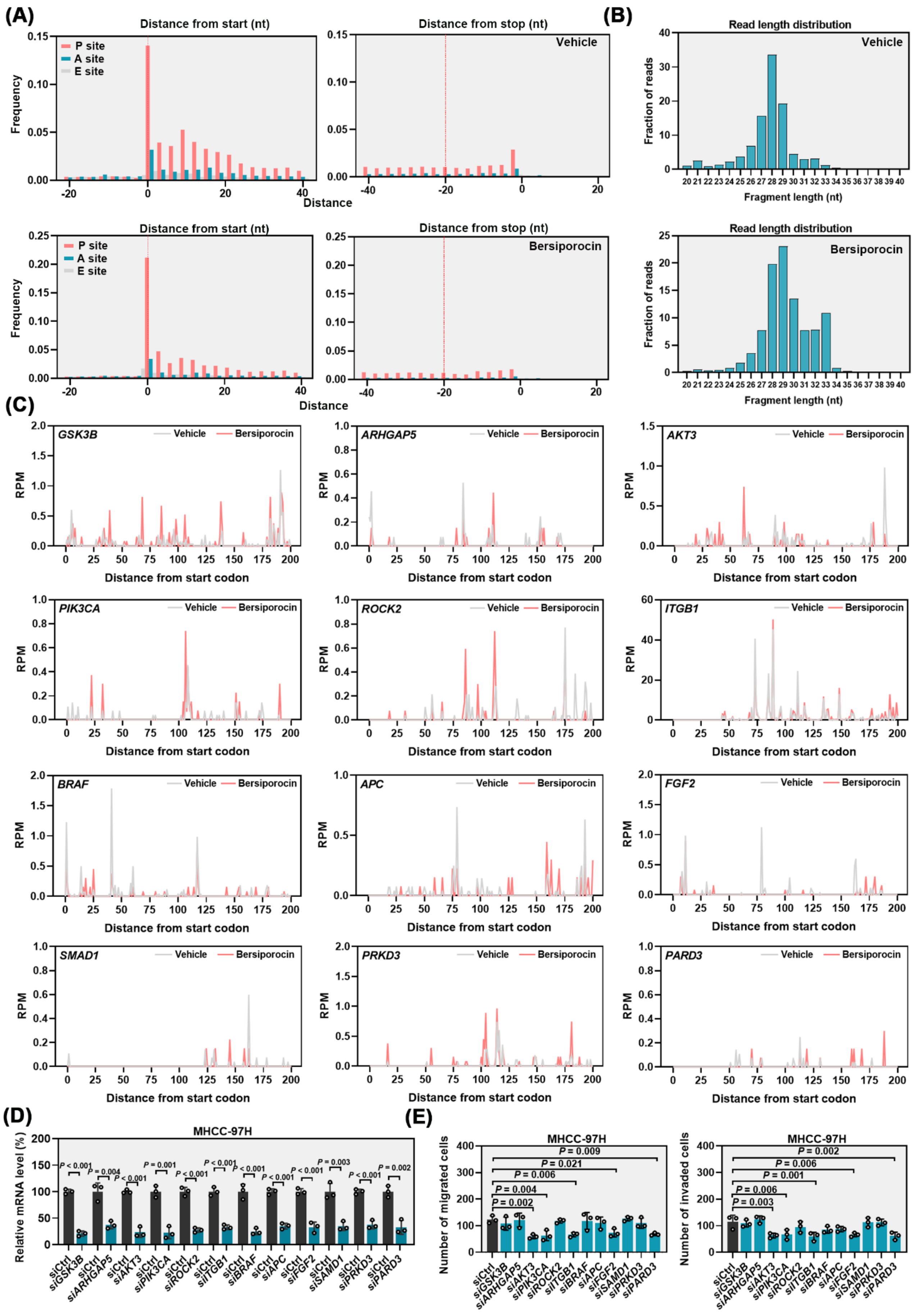

Supplementary Figure S6. Translational landscape of bersiporocin-treated HCC cells.

(A) The Bar charts showing the frequency of ribosome-protected fragment, illustrating the 3-nucleotide periodicity in the vehicle control and bersiporocin-treated groups (10 μmol/L, 24 h; *n* = 3 per group). **(B)** Bar chart showing the distribution of RPF lengths (20-40 nt) in the indicated treatment groups. **(C)** Peak plots showing the distribution of ribosome footprint occupancy at the A-site along the coding regions of *GSK3B*, *ARHGAP5*, *AKT3*, *PIK3CA*, *ROCK2*, *ITGB1*, *BRAF*, *APC*, *FGF2*, *SMAD1*, *PRKD3* and *PARD3* mRNAs in the indicated treatment groups. **(D)** RT-qPCR analysis of the relative mRNA levels in MHCC-97H cells respectively transfected with siCtrl, *siGSK3B*, *siARHGAP5*, *siAKT3*, *siPIK3CA*, *siROCK2, siITGB1*, *siBRAF*, *siAPC*, *siFGF2*, *siSMAD1*, *siPRKD3* and *siPARD3* (*n* = 3 per group). (**E**) Transwell assays showing the migratory and invasive abilities in MHCC-97H cells from the indicated treatment conditions in (D; *n* = 3 per group). Data are presented as mean ± SD, and *P* values were calculated using Student's *t*-test (D) and one-way ANOVA (E). Abbreviations: AKT3, AKT serine/threonine kinase 3; APC, adenomatous polyposis coli; ARHGAP5, Rho GTPase activating protein 5; BRAF, b-raf proto-oncogene, serine/threonine kinase; Ctrl, control; FGF2, fibroblast growth factor 2; GSK3B, glycogen synthase kinase 3 beta; ITGB1, integrin subunit beta 1; PARD3, par-3 family cell polarity regulator; PIK3CA, phosphatidylinositol-4,5-bisphosphate 3-kinase catalytic subunit alpha; PRKD3, protein kinase d3; Ribo-seq, ribosome profiling; ROCK2, Rho associated coiled-coil containing protein kinase 2; RPF, ribosome protected fragments; RPM, reads per million; RT-qPCR, quantitative fluorescence PCR; SMAD1, SMAD family member 1.

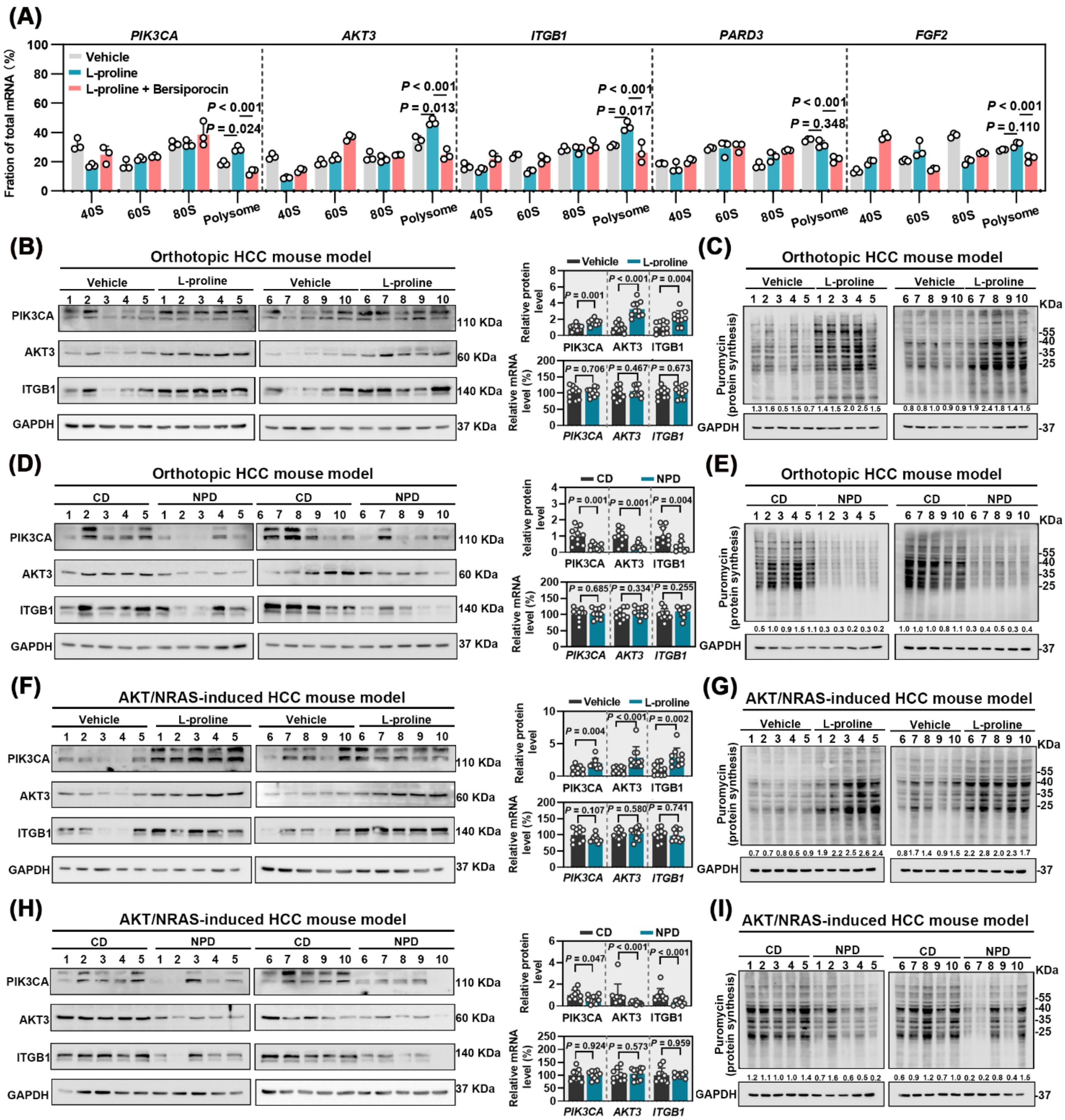

Supplementary Figure S7. The translation efficiency of PIK3CA, AKT3 and ITGB1.

(A) RT-qPCR analysis of the relative mRNA distributions of *PIK3CA*, *AKT3*, *ITGB1*, *PARD3,* and *FGF2* in ribosome fractions of MHCC-97H cells following treatment with L-proline (1 mmol/L, 24h) or the combination of L-proline and bersiporocin (10 μmol/L, 24h; *n* = 3 per group). (**B**) Western blotting and RT-qPCR, respectively, show the protein levels and mRNA levels of PIK3CA, AKT3, and ITGB1 in tumor tissues from orthotopic HCC mouse models. Mice were treated intraperitoneally every 2 days with either vehicle (PBS) or L-proline (500 mg/kg) for 5 weeks (*n* = 10 per group). (**C**) Western blotting showing the protein levels of puromycin incorporation in tumor cells from orthotopic HCC mouse models from the indicated treatment conditions in (B). (**D**) Western blotting and RT-qPCR, respectively, show the protein

levels and mRNA levels of PIK3CA, AKT3, and ITGB1 in tumor tissues from orthotopic HCC mouse models. Mice were fed with either CD or NPD (*n* = 10 per group) for 5 weeks. (**E**) Western blotting showing the protein levels of puromycin incorporation in tumor cells from orthotopic HCC mouse models from the indicated treatment conditions in (D). (**F**) Western blotting and RT-qPCR, respectively, show the protein levels and mRNA levels of PIK3CA, AKT3, and ITGB1 in tumor tissues from AKT/NRAS-induced HCC mouse models. Mice were treated intraperitoneally every 2 days with either vehicle (PBS) or L-proline (500 mg/kg) for 4 weeks (*n* = 10 per group). (**G**) Western blotting showing the protein levels of puromycin incorporation in tumor cells from AKT/NRAS-induced HCC mouse models from the indicated treatment conditions in (F). (**H**) Western blotting and RT-qPCR, respectively, show the protein levels and mRNA levels of PIK3CA, AKT3, and ITGB1 in tumor tissues from AKT/NRAS-induced HCC mouse models. Mice were fed with either CD or NPD (*n* = 10 per group) for 4 weeks. (**I**) Western blotting showing the protein levels of puromycin incorporation in tumor cells from AKT/NRAS-induced HCC mouse models from the indicated treatment conditions in (H). GAPDH was used as a loading control (B-I). Data are presented as mean ± SD, and *P* values were calculated using two-way ANOVA (A) and Student's *t*-test (B, D, F, H). Abbreviations: AKT, AKT serine/threonine kinase; AKT3, AKT serine/threonine kinase 3; CD, control diet; FGF2, fibroblast growth factor 2; GAPDH , glyceraldehyde-3-phosphate dehydrogenase; HCC, hepatocellular carcinoma; ITGB1, integrin subunit beta 1; NPD, proline-free diet; NRAS, neuroblastoma RAS viral oncogene homolog; PARD3, par-3 family cell polarity regulator; PBS, phosphate-buffered saline; PIK3CA, phosphatidylinositol-4,5-bisphosphate 3-kinase catalytic subunit alpha; RT-qPCR, quantitative fluorescence PCR.

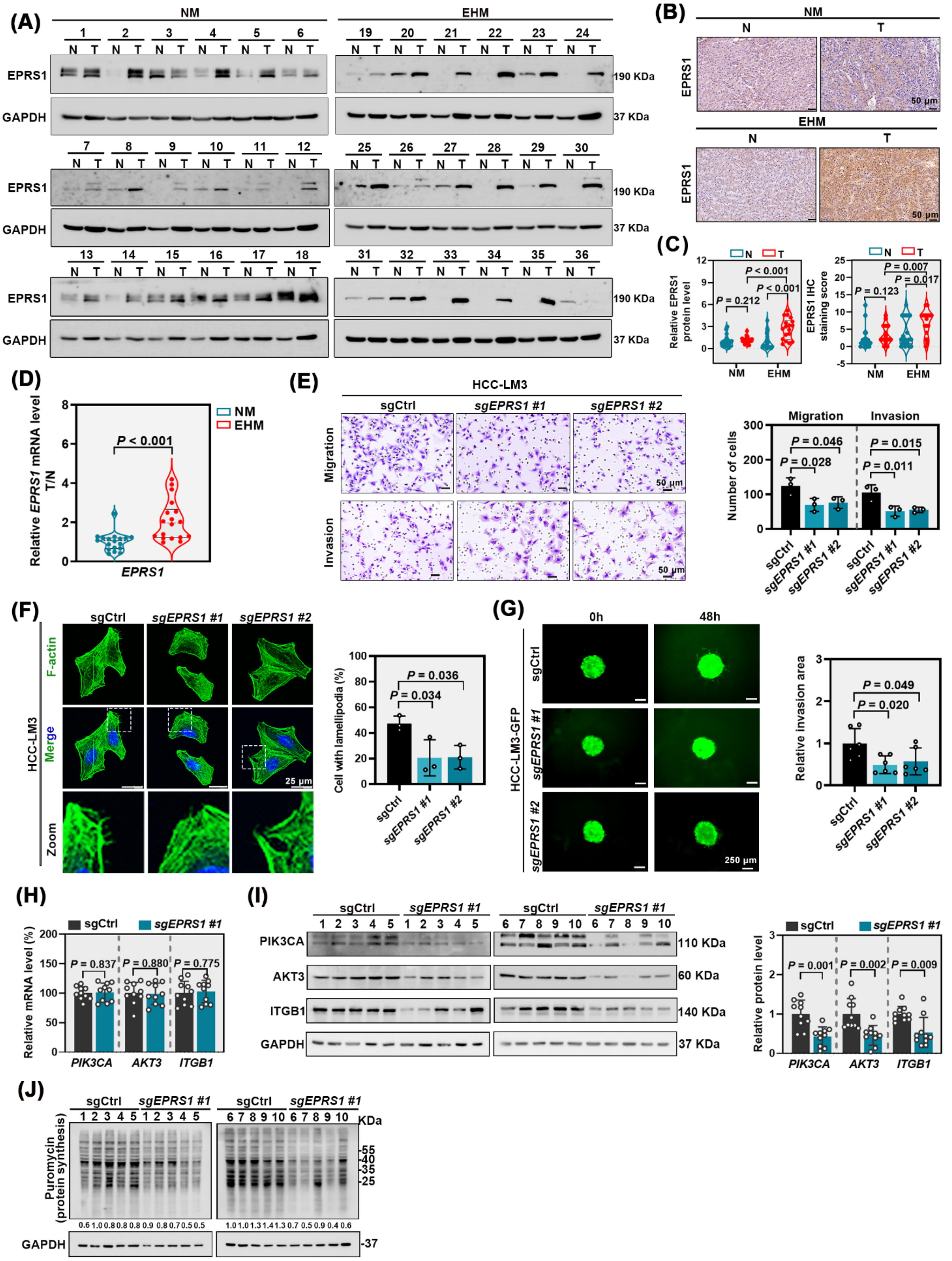

Supplementary Figure S8. The expression of EPRS1 in HCC tissues and EPRS1-induced HCC metastasis.

(A-C) Left, representative images of Western blotting (A) and IHC (B) showing the protein levels of EPRS1 in paired

tumor and adjacent normal tissues from HCC patients in cohort 1 (*n* = 36). The quantification data was shown (C). **(D)** RT-qPCR analysis of the mRNA level of *EPRS1* in paired tumor and adjacent normal tissues from HCC patients in cohort 1 (*n* = 36). T/N represents primary HCC tissues/adjacent non-tumor liver tissue. (**E**) Transwell assays showing the migratory and invasive abilities of control (sgCtrl) or *EPRS1*-knockout (*sgEPRS1 #1* and *sgEPRS1 #2*) HCC-LM3 cells. The quantification data was shown on the right (*n* = 3 per group). (**F**) Left, representative immunofluorescence images of F-actin organization in sgCtrl*, sgEPRS1 #1* and *sgEPRS1 #2* HCC-LM3 cells. Cells were stained with phalloidin (green, F-actin) and DAPI (blue, nuclei). The quantification data was shown on the right (*n* = 3 per group). (**G)** Left, representative images showing the *in vitro* 3D invasion in sgCtrl*, sgEPRS1 #1* and *sgEPRS1 #2* HCC-LM3-GFP cells. The quantification data was shown on the right (*n* = 6 per group). Images were captured at 0 hours and 48 hours after the spheroids were embedded into the invasion matrix. (**H-J**) sgCtrl *or sgEPRS1 #1* MHCC-97H cells were orthotopically injected into the BALB/c nude mice (*n* = 10 per group). RT-qPCR (H) and Western blotting (I) respectively showing the mRNA levels and protein levels of PIK3CA, AKT3, and ITGB1 in liver tumor tissues (*n* = 10 per group). (J) Western blotting showing the protein levels of puromycin incorporation in liver tumor cells (*n* = 10 per group). GAPDH was used as a loading control (A, I-J). Data are presented as mean ± SD, and *P* values were calculated using non-parametric t-tests (C-D), one-way ANOVA (E-G) and Student's *t*-test (H-I). Abbreviations: AKT3, AKT serine/threonine kinase 3; DAPI, 4',6-Diamidino-2-Phenylindole; EHM, extrahepatic metastasis; EPRS1, glutamyl-prolyl-tRNA synthetase 1; GAPDH, glyceraldehyde-3-phosphate dehydrogenase; HCC, hepatocellular carcinoma; IHC, immunohistochemistry; ITGB1, integrin subunit beta 1; N, adjacent non-tumor liver tissue; NM, no metastasis; PIK3CA, phosphatidylinositol-4,5-bisphosphate 3-kinase catalytic subunit alpha; RT-qPCR, quantitative fluorescence PCR; T, primary HCC tissues.

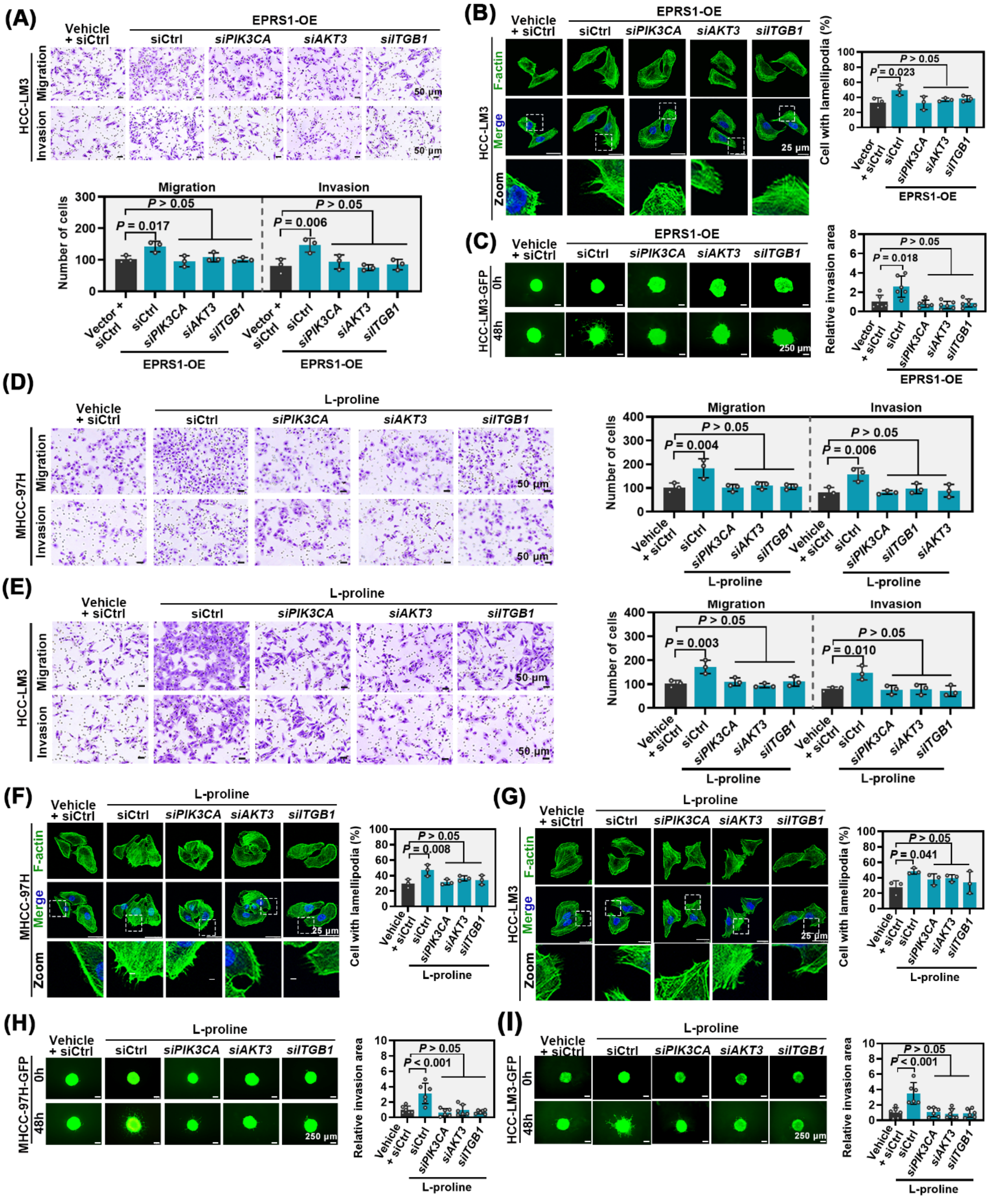

Supplementary Figure S9. PIK3CA, AKT3, and ITGB1 mediated the metastasis-related phenotypes in an L-proline and EPRS1-dependent manner

(**A**) Transwell assays showing the migratory and invasive abilities of HCC-LM3 cells under different treatment

conditions (vector + siCtrl, transfected with vector plasmid and siCtrl; EPRS1-OE + siCtrl, transfected with Flag-EPRS1 plasmid and siCtrl; EPRS1-OE + *siPIK3CA*, transfected with Flag-EPRS1 plasmid and *siPIK3CA*; EPRS1-OE + *siAKT3*, transfected with Flag-EPRS1 plasmid and *siAKT3*; EPRS1-OE + *siITGB1*, transfected with Flag-EPRS1 plasmid and *siITGB1*). The quantification data were shown on the lower panel (n = 3 per group). (**B**) Left, representative immunofluorescence images of F-actin organization from the indicated treatment conditions in (A). HCC-LM3 cells were stained with phalloidin (green, F-actin) and DAPI (blue, nuclei). The quantification data was shown on the right (n = 3 per group). (**C**) Left, representative images showing the *in vitro* 3D invasion of HCC-LM3-GFP cells from the indicated treatment conditions in (A). The quantification data was shown on the right (n = 6 per group). Images were captured at 0 hours and 48 hours after the spheroids were embedded into the invasion matrix. (**D-E**) Transwell assays showing the migratory and invasive abilities of MHCC-97H (D) and HCC-LM3 (E) cells under different treatment conditions (Vehicle + siCtrl, treated with PBS and transfected with siCtrl; L-proline+ siCtrl, treated with L-proline and transfected with siCtrl; L-proline + *siPIK3CA*, treated with L-proline and transfected with *siPIK3CA*; L-proline + *siAKT3*, treated with L-proline and transfected with *siAKT3*; L-proline + *siITGB1*, treated with L-proline and transfected with *siITGB1*). L-proline was used at a concentration of 1 mmol/L for 24 h. The quantification data was shown on the lower panel (n = 3 per group). (**F-G**) Left, representative immunofluorescence images of F-actin organization from the indicated treatment conditions in (D). MHCC-97H (F) and HCC-LM3 cells (G) were stained with phalloidin (green, F-actin) and DAPI (blue, nuclei). The quantification data were shown on the right (n = 3 per group). (**H-I**) Left, representative images showing the *in vitro* 3D invasion of MHCC-97H-GFP (H) and HCC-LM3-GFP cells (I) from the indicated treatment conditions in (D). The quantification data was shown on the right (n = 6 per group). Data are presented as mean ± SD, and P values were calculated using one-way ANOVA (A-I). Abbreviations: AKT3, AKT serine/threonine kinase 3; Ctrl, control; DAPI, 4',6-Diamidino-2-Phenylindole; EPRS1, glutamyl-prolyl-tRNA synthetase 1; ITGB1, integrin subunit beta 1; OE, overexpression; PIK3CA, phosphatidylinositol-4,5-bisphosphate 3-kinase catalytic subunit alpha.

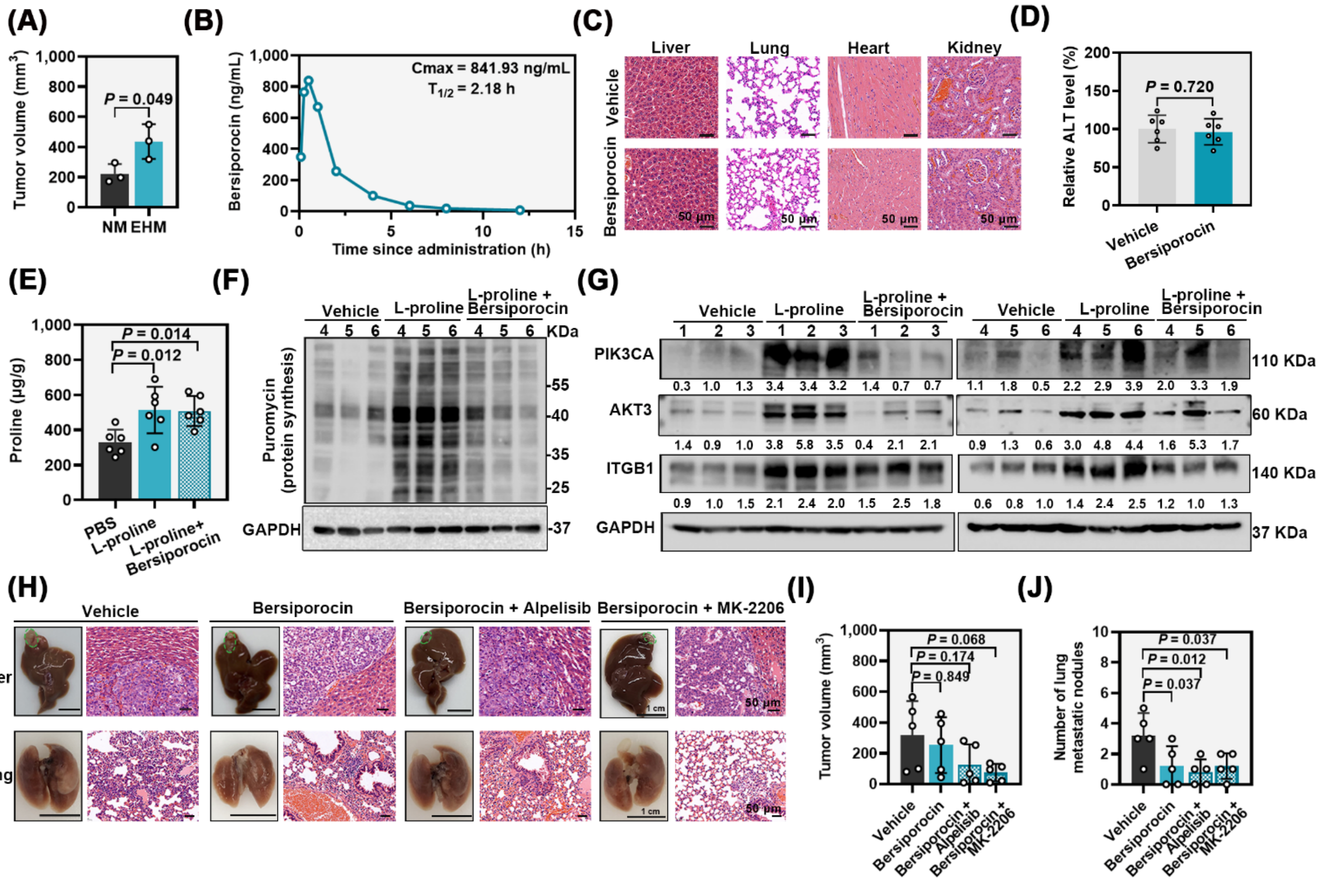

Supplementary Figure S10. Pharmacodynamic profiling and anti-metastasis efficacy of bersiporocin.

(A) Bar charts showing the liver tumor volume of NM-derived and EHM-derived PDOX models (*n* = 3 per group). **(B)** Plasma concentration-time curve of bersiporocin (10 mg/kg) following a single intraperitoneal administration to C57BL/6J mice. Blood samples were collected at indicated time points (0-12 h), and bersiporocin levels were quantified by LC-MS/MS (*n* = 3 per group). (**C**) Representative H&E staining images of liver, lung, heart, and kidney tissues from C57BL/6J mice treated with vehicle (DMSO in PBS) or bersiporocin (10 mg/kg; *n* = 6 per group). (**D**) Bar chart showing the serum ALT level detected in C57BL/6J mice from the indicated treatment groups. (**E**) Bar charts showing the proline levels quantified by a colorimetric assay in liver tumor tissues from EHM-derived PDOX models treated with vehicle (DMSO in PBS), L-proline (500 mg/kg), or the combination of L-proline and bersiporocin (10 mg/kg; *n* = 6 per group). (**F-G**) Western blotting showing the protein levels of puromycin incorporation (F), and PIK3CA, AKT3, ITGB1 (G) in tumor tissues from EHM-derived PDOX models from the indicated treatment conditions in (E). (**H-J**) EHM-derived PDOX mice were administered vehicle (DMSO in PBS), bersiporocin (10 mg/kg), combination of bersiporocin and Alpelisib (25 mg/kg), or combination of bersiporocin and MK-2206 (100 mg/kg; *n* = 5 per group). Representative images of liver and lung, and corresponding H&E staining, were shown (H). Bar charts showing the liver tumor volume (I) and number of lung metastatic nodules (J) from the indicated treatment groups in (H). GAPDH was used as a loading

control (F-G). Data are presented as mean ± SD, and *P* values were calculated using Student's *t*-test (A, D) and one-way ANOVA (E, I-J). Abbreviations: AKT3, AKT serine/threonine kinase 3; ALT, alanine aminotransferase; EHM, extrahepatic metastasis; GAPDH, glyceraldehyde-3-phosphate dehydrogenase; HCC, hepatocellular carcinoma; ITGB1, integrin subunit beta 1; NM, no metastasis; PBS, phosphate-buffered saline; PDOX, patient-derived orthotopic xenograft; PIK3CA, phosphatidylinositol-4,5-bisphosphate 3-kinase catalytic subunit alpha; TNM, tumor node metastasis.

Supplementary Table S1. The clinical characteristics of enrolled individuals in cohort 1.

**Characteristics**	**HCC (*n* = 91)**	**Control (*n* = 20)**	***P* value**
Gender, *n* (%)			0.078
Male	73 (80.22)	12 (60.00)	
Female	18 (19.78)	8 (40.00)	
Age, years, *n* (%)			0.280
<60	53 (58.24)	9 (45.00)	
≥60	38 (41.76)	11 (55.00)	
Etiology, *n* (%)			N/A
Viral	42 (46.15)	0 (0.00)	
Non-viral	49 (53.85)	20 (100.00)	
TNM stage, *n* (%)			N/A
I	19 (20.88)	N/A	
II	16 (17.58)	N/A	
III	15 (16.48)	N/A	
IV	41 (45.05)	N/A	
Site of metastasis, *n* (%)			N/A
None	50 (54.95)	20 (100.00)	
Lung only	25 (27.47)	N/A	
Bone only	8 (8.79)	N/A	
Distant lymph nodes only	6 (6.59)	N/A	
Multiple metastases	2 (2.20)	N/A	

P values were calculated using the chi-squared test or Fisher's exact test.

Abbreviations: HCC, hepatocellular carcinoma; N/A, not applicable; TNM, tumor node metastasis.

Supplementary Table S2. The clinical characteristics of patients with paired tumors and adjacent normal tissues in cohort 1.

Characteristics	NM (n = 18)	EHM (n = 18)	P value
Gender, n (%)			0.228
Male	12 (66.67)	16 (88.89)	
Female	6 (33.33)	2 (11.11)	
Age, years, n (%)			0.735
<60	10 (55.56)	11 (61.11)	
≥60	8 (44.44)	7 (38.89)	
Etiology, n (%)			0.738
Viral	9 (50.00)	10 (55.56)	
Non-viral	9 (50.00)	8 (44.44)	
TNM stage, n (%)			N/A
I	8 (44.44)	N/A	
II	5 (27.78)	N/A	
III	5 (27.78)	N/A	
IV	N/A	18 (100.00)	
Site of metastasis, n (%)			N/A
None	18 (100.00)	N/A	
Lung only	N/A	11 (61.11)	
Bone only	N/A	3 (16.67)	
Distant lymph nodes only	N/A	2 (11.11)	
Multiple metastases	N/A	2 (11.11)	

P values were calculated using the chi-squared test or Fisher's exact test.

Abbreviations: EHM, extrahepatic metastasis; NM, no metastasis; N/A, not applicable; TNM, tumor node metastasis.

Supplementary Table S3. The clinical characteristics of patients with tumor tissue subjected to RNA-seq in cohort 1.

**Characteristics**	**NM** **(*n* = 9)**	**EHM** **(*n* = 8)**	***P* value**
Gender, *n* (%)			0.576
Male	6 (66.67)	7 (87.50)	
Female	3 (33.33)	1 (12.50)	
Age, years, *n* (%)			0.637
<60	6 (66.67)	4 (50.00)	
≥60	3 (33.33)	4 (50.00)	
Etiology, *n* (%)			0.637
Viral	4 (44.44)	5 (62.50)	
Non-viral	5 (55.56)	3 (37.50)	
TNM stage, *n* (%)			N/A
I	5 (55.56)	N/A	
II	2 (22.22)	N/A	
III	2 (22.22)	N/A	
IV	N/A	8 (100.00)	
Site of metastasis, *n* (%)			N/A
None	9 (100.00)	N/A	
Lung only	N/A	3 (37.50)	
Bone only	N/A	2 (25.00)	
Distant lymph nodes only	N/A	2 (25.00)	
Multiple metastases	N/A	1 (12.50)	

P values were calculated using the chi-squared test or Fisher's exact test.

Abbreviations: EHM, extrahepatic metastasis; NM, no metastasis; N/A, not applicable; TNM, tumor node metastasis.

Supplementary Table S4. The clinical characteristics of enrolled individuals in cohort 2.

Characteristics	HCC (n = 176)	Control (n = 52)	P value
Gender, n (%)			0.800
Male	105 (59.66)	30 (57.69)	
Female	71 (40.34)	22 (42.31)	
Age, years, n (%)			0.300
<60	82 (46.59)	20 (38.46)	
≥60	94 (53.41)	32 (61.54)	
Etiology, n (%)			N/A
Viral	106 (60.23)	0 (0.00)	
Non-viral	70 (39.77)	52 (100.00)	
TNM stage, n (%)			N/A
I	77 (43.75)	N/A	
II	12 (6.82)	N/A	
III	27 (15.34)	N/A	
IV	60 (34.09)	N/A	
Site of metastasis, n (%)			N/A
None	116 (65.91)	52 (100.00)	
Lung only	32 (18.18)	N/A	
Bone only	13 (7.39)	N/A	
Distant lymph nodes only	9 (5.11)	N/A	
Multiple metastases	6 (3.41)	N/A	

P values were calculated using the chi-squared test or Fisher's exact test.

Abbreviations: HCC, hepatocellular carcinoma; N/A, not applicable; TNM, tumor node metastasis.

Supplementary Table S5. Primers for RT-qPCR assays.

Name	Sequence (5' to 3')	Supplier
(Human) ACACA-F	CTCTTGGCCTTTTCCCGGTC	Beijing Tsingke
(Human) ACACA-R	GCCCTCCTTCTCCTCCAGTA	Beijing Tsingke
(Human) AKT3-F	TTTTCTCTATTATTTGGGCTGAGTC	Beijing Tsingke
(Human) AKT3-R	ACCATAGAAACGTGTGCGGT	Beijing Tsingke
(Human) ALDH4A1-F	GAATGTGCTGCCCTTTCAGC	Beijing Tsingke
(Human) ALDH4A1-R	TCCACCGCATACTTGGCATT	Beijing Tsingke
(Human) APC-F	AGCAAGTTGAGGCACTGAAGA	Beijing Tsingke
(Human) APC-R	ACCGCAGTTTTACTCCAGGG	Beijing Tsingke
(Human) ARG1-F	TGTGAAGAACCCACGGTCTG	Beijing Tsingke
(Human) ARG1-R	CCAGCACCACACTGACTCTT	Beijing Tsingke
(Human) ARHGAP5-F	AACTTGATCTTCGGCCGGTT	Beijing Tsingke
(Human) ARHGAP5-R	GCCACCAGCTGTACAGGAAT	Beijing Tsingke
(Human) BRAF-F	TCCGGAGGAGGTGTGGAATA	Beijing Tsingke
(Human) BRAF-R	TCTCTGCTAAGGACGCCTCT	Beijing Tsingke
(Human) EEF2-F	TCAAACTGGACAGCGAGGAC	Beijing Tsingke
(Human) EEF2-R	TCGTACTTCTCGGCCAGGTA	Beijing Tsingke
(Human) EPRS1-F	AACTCCGACCAAACAAGGTG	Beijing Tsingke
(Human) EPRS1-R	TGTGGGGAAATTGACTGTGA	Beijing Tsingke
(Human) FGF2-F	ACCGAAATGCTGGAGGTGTT	Beijing Tsingke
(Human) FGF2-R	AGCCCAGTTAGAGGGACCAT	Beijing Tsingke
(Human) FLNA-F	CTGCTCGGTCGAGTACATCC	Beijing Tsingke
(Human) FLNA-R	CGTGACCTCCGATTGACACT	Beijing Tsingke
(Human) GSK3B-F	TCCATTCCTTTGGAATCTGC	Beijing Tsingke
(Human) GSK3B-R	CAATTCAGCCAACACACACAGC	Beijing Tsingke
(Human) HSPD1-F	GACGACCTGTCTCGCCG	Beijing Tsingke
(Human) HSPD1-R	GGACTTCCCCAACTCTGCTC	Beijing Tsingke
(Human) IQGAP1-F	TTCGCCACTACCCAGACCTTGTTT	Beijing Tsingke
(Human) IQGAP1-R	CCTGTCTTGGATGTGGCCTTTGG	Beijing Tsingke

(Human) ITGB1-F	CGCCGCGCGGAAAAGATG	Beijing Tsingke
(Human) ITGB1-R	AAACACCAGCAGCCGTGTAA	Beijing Tsingke
(Human) OAT-F	CGCTGTCAGATCTGTGGTTTTTC	Beijing Tsingke
(Human) OAT-R	GGTGACAATGCCCTTGGTTG	Beijing Tsingke
(Human) P4HA1-F	GAGTATCTAAGAGTGCCTGGCT	Beijing Tsingke
(Human) P4HA1-R	CGTGCAAAGTCAAAATGGGGT	Beijing Tsingke
(Human) P5CS-F	ATTGGAGTTCTGCTGGTGATCTT	Beijing Tsingke
(Human) P5CS-R	GCGGCAAAGATCTTCAACTTCTT	Beijing Tsingke
(Human) PARD3-F	TACCAGCTGTCCCCTACAGT	Beijing Tsingke
(Human) PARD3-R	AAGTACCAGCATCTGCCGTC	Beijing Tsingke
(Human) PIK3CA-F	CCACGACCATCATCAGGTGAA	Beijing Tsingke
(Human) PIK3CA-R	CCTCACGGAGGCATTCTAAAGT	Beijing Tsingke
(Human) PRKD3-F	GAGCCTGCCACTGCTAACTA	Beijing Tsingke
(Human) PRKD3-R	GTCCTCATTTTCATTCTGGGGG	Beijing Tsingke
(Human) PRODH-F	GAGGAGCTACAGATGACCAGGAT	Beijing Tsingke
(Human) PRODH-R	ACATTGAACTTCCGCTGCATCTC	Beijing Tsingke
(Human) PYCR1-F	CGACATTGAGGACAGACACATTG	Beijing Tsingke
(Human) PYCR1-R	ACGACTGGAGTGTTGGTCATG	Beijing Tsingke
(Human) PYCR2-F	TCCCCAGAAATGAACCTGCC	Beijing Tsingke
(Human) PYCR2-R	AGCCATGAATGCCTTCTCCA	Beijing Tsingke
(Human) PYCR3-F	GGAGGAGGAAGGGGCCATA	Beijing Tsingke
(Human) PYCR3-R	TCGGAGAATGCACACACGAA	Beijing Tsingke
(Human) ROCK2-F	TCCCGATAACCACCCCTCTT	Beijing Tsingke
(Human) ROCK2-R	GGAAAAAGGCTTTCCAGCCG	Beijing Tsingke
(Human) SLC36A4-F	TGGACCAATCAGCCTTGTGTTTA	Beijing Tsingke
(Human) SLC36A4-R	GCAAAGCTCACAGTGTCACTATAA	Beijing Tsingke
(Human) SLC38A1-F	GCTTTGGTTAAAGAGCGGGC	Beijing Tsingke
(Human) SLC38A1-R	TACGAACTTCCCTGTGGTGC	Beijing Tsingke
(Human) SLC38A2-F	GCCTTAGAACGCCTTTCCAG	Beijing Tsingke
(Human) SLC38A2-R	AATCGTCCCATTTCGGCCTT	Beijing Tsingke

(Human) SLC38A4-F	CCCCACTCACACAGAACAGAG	Beijing Tsingke
(Human) SLC38A4-R	CAGCGCTTTCTTGTCCACAC	Beijing Tsingke
(Human) SMAD1-F	GCTCCCTGTCTTTGTGCTGAC	Beijing Tsingke
(Human) SMAD1-R	GTCTCTTCACAGCTGGACTTGTA	Beijing Tsingke
(Human) β-actin-F	CTCTTCCAGCCTTCCTTCCT	Beijing Tsingke
(Human) β-actin-R	AGCACTGTGTTGGCGTACAG	Beijing Tsingke
(Mus) Akt3- F	CATCTGAAACAGACACCCGATA	Beijing Tsingke
(Mus) Akt3- R	GTCCGCTTGCAGAGTAGGAG	Beijing Tsingke
(Mus) Itgb1- F	AACTTGTTGGTCAGCAACGC	Beijing Tsingke
(Mus) Itgb1- R	AGCCAATCAGCGATCCACAA	Beijing Tsingke
(Mus) Pik3ca- F	GAAAATGGCTTTGAATCTCTGG	Beijing Tsingke
(Mus) Pik3ca- R	GATACATCCCACAGGCACG	Beijing Tsingke
(Mus) β-actin-F	AGAAGATCTGGCACCACACC	Beijing Tsingke
(Mus) β-actin-R	TACGACCAGAGGCATACAGG	Beijing Tsingke

Abbreviations: ACACA, acetyl-CoA carboxylase alpha; AKT3, AKT serine/threonine kinase 3; ALDH4A1, aldehyde dehydrogenase 4 family member A1; APC, adenomatous polyposis coli; ARG1, arginase 1; ARHGAP5, Rho GTPase activating protein 5; BRAF, B-Raf proto-oncogene, serine/threonine kinase; EEF2, eukaryotic translation elongation factor 2; EPRS1, glutamyl-prolyl-tRNA synthetase 1; FGF2, fibroblast growth factor 2; FLNA, filamin A; GSK3B, glycogen synthase kinase 3 beta; HSPD1, heat shock protein family D member 1; IQGAP1, IQ motif containing GTPase activating protein 1; ITGB1, integrin subunit beta 1; OAT, ornithine aminotransferase; P4HA1, prolyl 4-hydroxylase subunit alpha 1; P5CS, pyrroline-5-carboxylate synthase; PARD3, par-3 family cell polarity regulator; PIK3CA, phosphatidylinositol-4,5-bisphosphate 3-kinase catalytic subunit alpha; PRKD3, protein kinase D3; PRODH, proline dehydrogenase; PYCR1, pyrroline-5-carboxylate reductase 1; PYCR2, pyrroline-5-carboxylate reductase 2; PYCR3, pyrroline-5-carboxylate reductase 3; ROCK2, Rho associated coiled-coil containing protein kinase 2; SLC36A4, solute carrier family 36 member 4; SLC38A1, solute carrier family 38 member 1; SLC38A2, solute carrier family 38 member 2; SLC38A4, solute carrier family 38 member 4; SMAD1, SMAD family member 1.

Supplementary Table S6. Antibodies, reagents, and resources used in this study.

Name	Application	Dilution	Supplier	Cat No.
anti-Flag	Western blotting	1:2,000	Sigma-Aldrich	F1804
anti-GAPDH	Western blotting	1: 1,000	Santa Cruz Biotechnology	sc-47724
anti-puromycin	Western blotting	1:2,000	Merck	MABE343
anti-PIK3CA	Western blotting	1:1,000	HUABIO	ET1606-36
anti-AKT3	Western blotting	1:1,000	Wanleibio	WL0005a
anti-ITGB1	Western blotting	1:1,000	HUABIO	ET1601-17
anti-EPRS1	Western blotting, immunocytochemistry, Immunofluorescence	1:1,000 (WB), 1:500 (IHC), 1:100 (IF)	Proteintech	67712-1-Ig
anti-P5CS	Western blotting, immunohistochemistry, multiplex immunofluorescence staining	1:1,000 (WB), 1:500 (IHC), 1:200 (mIF)	Proteintech	17719-1-AP
anti-PYCR1	Western blotting, immunohistochemistry, multiplex immunofluorescence staining	1:1,000 (WB), 1:500 (IHC), 1:200 (mIF)	Proteintech	13108-1-AP
anti-PRODH	Western blotting, immunohistochemistry, multiplex immunofluorescence staining	1:1,000 (WB), 1:500 (IHC), 1:200 (mIF)	Proteintech	22980-1-AP
anti-FLNA	Western blotting	1:1,000	HUABIO	ET1601-3
anti-FASN	Western blotting	1:1,000	HUABIO	ET1701-91
anti-IQGAP1	Western blotting	1:1,000	HUABIO	ET7108-79
anti-GCN1	Western blotting	1:1,000	Abiowell	AWA67601
anti-PRKDC	Western blotting	1:5,000	HUABIO	ET1610-12
anti-EEF2	Western blotting	1:1,000	HUABIO	HA721349
anti-ACACA	Western blotting	1:1,000	HUABIO	ET1609-77
anti-HSPD1	Western blotting	1:20,000	HUABIO	ET1609-45
anti-ND1	Western blotting	1:1,000	HUABIO	HA721595
anti-CYTB	Western blotting	1:1,000	HUABIO	HA720084-10
anti-COX1	Western blotting	1:1,000	HUABIO	ET1610-98-10

anti-ATP6	Western blotting	1:1,000	Proteintech	55313-1-AP
Alpelisib	PDOX	25 mg/kg	TargetMoI	T1921
MK-2206	PDOX	100 mg/kg	TargetMoI	T1952
Isoleucine	Transwell	105 mg/L	Sigma-Aldrich	I2752
Methionine	Transwell	30 mg/L	Sigma-Aldrich	M5308
Histidine	Transwell	42 mg/L	Sigma-Aldrich	H8000
Alanine	Transwell	8.9 mg/L	Sigma-Aldrich	A7469
Serine	Transwell	52.5 mg/L	Sigma-Aldrich	S4311
Threonine	Transwell	95 mg/L	Sigma-Aldrich	T8625
Coll-I	Transwell	0.2 μg/mL	Southern Biotech	1200-01S
NADPH	Transwell	1 mmol/L	Merck	2646-71-1
Glutamine	Transwell	4 mmol/L	Invitrogen	A2916801
L-proline	Transwell, cell culture	1 mmol/L	Sigma-Aldrich	#P5607
All amino acids depleted DMEM medium	Cell culture	N/A	Yuchun Biology	YC-2082

Abbreviations: ACACA, acetyl-CoA carboxylase alpha; AKT3, AKT serine/threonine kinase 3; ATP6, ATP synthase F0 subunit 6; Coll-I, collagen type I; COX1, cytochrome c oxidase subunit I; CYTB, cytochrome b; DMEM, Dulbecco's modified Eagle's medium.; EEF2, eukaryotic translation elongation factor 2; EPRS1, glutamyl-prolyl-tRNA synthetase 1; FASN, fatty acid synthase; FLNA, filamin A; GAPDH, glyceraldehyde-3-phosphate dehydrogenase; GCN1, general control non-derepressible protein 1; HSPD1, heat shock protein family D member 1; IF, immunofluorescence; IHC, immunohistochemistry; IQGAP1, IQ motif containing GTPase activating protein 1; ITGB1, integrin subunit beta 1; mIF, multiplex immunofluorescence staining; NADPH, nicotinamide adenine dinucleotide phosphate; N/A, not applicable; ND1, NADH dehydrogenase subunit 1; P5CS, pyrroline-5-carboxylate synthase; PIK3CA, phosphatidylinositol-4,5-bisphosphate 3-kinase catalytic subunit alpha; PRKDC, protein kinase, DNA-activated, catalytic subunit; PRODH, proline dehydrogenase; PYCR1, pyrroline-5-carboxylate reductase 1; WB, Western blotting.

Supplementary Table S7. Clinical characteristics of the donor patients in HCC PDOX models.

**Group**	**Patient number**	**Gender**	**Age (Y)**	**TNM stage**	**Site of metastasis**
NM	#1	Male	40 ≤ Y<60	II	None
NM	#2	Male	≥60	III	None
NM	#3	Female	≥60	II	None
EHM	#4	Male	≥60	IV	Lung
EHM	#5	Male	≥60	IV	Lung
EHM	#6	Male	≥60	IV	Bone

Abbreviations: EHM, extrahepatic metastasis; NM, no metastasis; TNM, tumor node metastasis.
